# Extrication following a motor vehicle collision: a consensus statement on behalf of The Faculty of Pre-hospital Care, Royal College of Surgeons of Edinburgh

**DOI:** 10.1186/s13049-024-01312-z

**Published:** 2025-01-06

**Authors:** Tim Nutbeam, Rob Fenwick, Charlotte Haldane, Caroline Leech, Emily Foote, Simon Todd, David Lockey

**Affiliations:** 1 IMPACT, Centre for Post-Collision Research Innovation and Translation, Exeter, UK; 2 University of Plymouth, Plymouth, UK; 3 Betsi Cadwaladr University Health Board, Wrexham Maelor Hospital, Croesnewedd Road, Wrexham, UK; 4 Faculty of Pre-Hospital Care, Royal College of Surgeons Edinburgh, Edinburgh, UK; 5 University Hospitals Coventry and Warwickshire NHS Trust, Clifford Bridge Road, Walsgrave, Coventry, CV2 2DX UK; 6 University Hospitals Plymouth NHS Trust, Plymouth, UK; 7 Dorset and Wiltshire Fire and Rescue Service/South Western Ambulance Service Trust (SWAST), Exeter, UK

**Keywords:** Road traffic injury, Extrication, Post-collision, Trauma

## Abstract

**Background:**

Road traffic injury is the leading cause of death among young people globally, with motor vehicle collisions often resulting in severe injuries and entrapment. Traditional extrication techniques focus on limiting movement to prevent spinal cord injuries, but recent findings from the EXIT project challenge this approach. This paper presents updated recommendations from the Faculty of Pre-Hospital Care (FPHC) that reflect the latest evidence on extrication practices.

**Methods:**

A systematic scoping review identified 170 relevant articles from 7083 records. Findings, together with EXIT project data, informed the development of 12 core and supplemental statements on extrication. In April 2024, 43 subject matter experts from diverse backgrounds participated in a consensus process. Statements were discussed, voted on, and synthesised into the updated statement, ratified by FPHC.

**Results:**

Consensus was achieved for all 12 statements, emphasising self-extrication as a preferred, primary approach, reducing extrication time, and moving away from absolute movement minimisation. The U-STEP OUT algorithm was endorsed as a decision-making tool. Key themes included interdisciplinary collaboration, use of operational and clinical decision aids, and enhanced training.

**Conclusions:**

This consensus statement marks a paradigm shift in extrication practice, moving away from traditional movement minimisation to a focus on time-sensitive, patient-centred care. The findings advocate for empowering both clinical and non-clinical responders and improving interdisciplinary training and communication. Further research is needed to assess the broader implementation of this statement and to explore the psychological impacts of entrapment and extrication on patients.

**Supplementary Information:**

The online version contains supplementary material available at 10.1186/s13049-024-01312-z.

## Background

Road traffic injury is the leading cause of death in children and young adults aged 5–29 years [1]. In addition to the 1.3 million road deaths per year, an additional 20–50 million people incur significant injury and often long-term disability [1]. Motor vehicle collision (MVC) is the leading cause of road traffic injury [1]. Following an MVC, up to 40% of patients will remain trapped in their vehicles (supplementary text 1). Extrication is the process of removing injured or potentially injured patients from motor vehicles following a collision.

Rescue service extrication techniques have evolved since the 1950s. This evolution has been facilitated by the production of faster, more powerful cutting and lifting equipment. However, in the last 70 years there has been little change in the fundamental tenet of extrication: that of absolute 'movement minimisation’ (supplementary text 1). This has influenced strategies, techniques and approaches that conceptually lead to absolute minimal spinal movement of the patient being extricated. Rescue service guidelines and firefighter manuals inform us that the core purpose of absolute movement minimisation is to minimise the frequency and severity of secondary spinal cord injury [2, 3].

Closer examination of the movement minimisation concept raises the following considerations. Firstly, absolute movement minimisation takes time; the longer an extrication takes, the longer a patient will remain trapped and the timeline between injury and clinical intervention will extend. Where there is time-critical injury, this may result in excess death and increased morbidity [4]. Secondly, the utility of current extrication techniques to deliver movement minimisation was, until recently, unclear. Recent analysis has challenged the assumption that standard rescue techniques achieve their central purpose of movement minimisation [5]. Finally, the origins and justification of movement minimisation as the central tenet of extrication practice are unclear (supplementary text 1). Importantly, there is no high-quality evidence to inform the use of the absolute movement minimisation approach.

In more recent years these paradigms have come under increasing challenge. Commonly cited statistics on spinal injuries caused by rescuer handling are unsubstantiated and lack identifiable origins (supplementary text 1). Recent reports on injuries and outcomes, the biomechanical performance of extrication techniques and patient experience provides additional evidence of the need to provide updated guidance [4–7].

The Faculty of Pre-Hospital Care (FPHC) of the Royal College of Surgeons of Edinburgh (RCSEd) produces ‘Consensus Guidance’ for areas of clinical and operational practice. The FPHC initially considered guidance in relation to Extrication in 2012; however, the evidence available at the time was not sufficient to recommend changes to accepted practice. More recently, significant contributions to evidence in relation to extrication and immediate post-collision care have occurred enabling further consideration by the consensus process [4–6, 8–12].

The aim of this paper is to report the FPHC consensus process and its outcomes.

## Methods

**Fig. 1:**
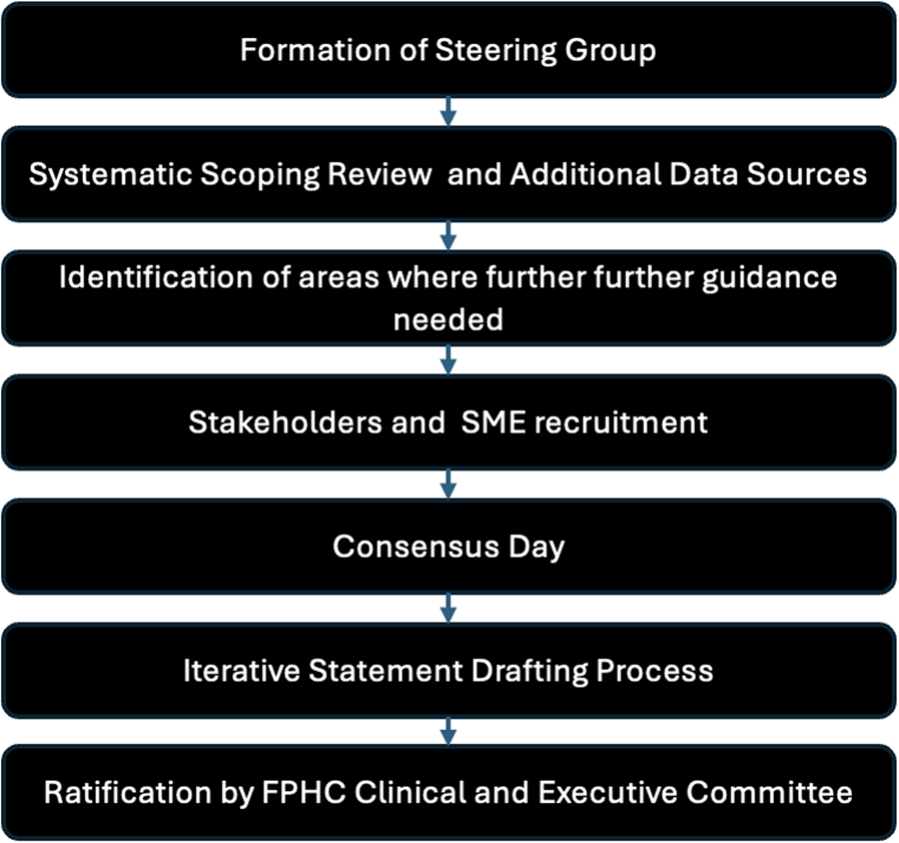
FPHC consensus statement process

The FPHC consensus process follows an established methodology for developing clinical guidance (Fig. 1).

The steering group comprised of established subject matter experts (SME) from Prehospital Emergency Medicine (PHEM), Emergency Medicine (EM) and the Fire and Rescue Service (FRS). The steering group were selected by the FPHC consensus lead (CH) and the lead author (TN). The steering group are the authors of the guidance and this paper.

The systematic scoping literature review considered 7089 articles of which 170 were included in the qualitative synthesis (supplementary text 1). Key themes within this review included extrication training and principles, injuries, immobilisation, care during entrapment, clinical response type, vehicle deformity intrusion entrapment, and tool-based extrication. This review was supplemented with the findings of the EXIT project (supplementary text 2). The steering group reviewed the available literature and identified areas where further guidance would offer clinical and operational advantage.

The steering group identified key stakeholder organisations and individual SMEs based on their significant influence on and active participation in UK extrication practice. Stakeholder organisations were invited to nominate SMEs from within their membership to attend the consensus-finding day, while individual experts with recognised expertise in relevant areas were directly invited to participate. This approach ensured that the consensus process included a diverse and representative group of contributors with substantial influence on UK extrication practice (Table 1). Literature reviews and additional areas for consideration were circulated to participants to allow them to prepare for the consensus day (supplementary text 3). SMEs were asked to submit suggestions for consideration at the consensus day. These statements/areas of clinical or operational practice were reviewed by the steering group, collapsed and duplicates removed.

**Table 1:** Organisations with SMEs at consensus day

Organisation and Number of SME representatives (n)	
British Association for Immediate Care (BASICs) Scotland (1)	Devon Somerset FRS (8)
BASICs (England) (1)	Dorset & Wiltshire FRS (1)
College of Paramedics (2)	Essex FRS (1)
Devon Air Ambulance (2)	Scottish FRS (3)
Northern Ireland Ambulance Service (1)	South East Ambulance Service (2)
Scottish Ambulance Service (SAS) (1)	South Western Ambulance Service (4)
Welsh Ambulance Service NHS Trust (2)	UKRO/NFCC (3)
Yorkshire Ambulance Service NHS Trust (1)	National Fire Chiefs Council (NFCC) (1)
Defence Medical Academy Project Packington (1)	FireWiseUK Learning Academy (1)
National Ambulance Resilience Unit (NARU) (1)	International Road Rescue & Trauma Consultancy (IRRTC) (1)
National HEMS Research and Audit Forum (NHRAF) (1)	Federation International Automobile (1)
ATACC (2)	College of Policing (1)

The Consensus meeting was held in-person on the 15th April 2024. Each of the statements was presented to the attending SMEs, verbal submissions were welcomed by the audience and discussion encouraged. When discussion concluded, SMEs voted anonymously from each other on the discussed statement using an online voting platform (Slido 2024, Bratislava, Slovakia). SMEs could vote agree, disagree or had the option to ‘opt out’ if the specific question was outside of their area of expertise. Consistent with previous studies, consensus was set a priori at 70% agreement or disagreement of participating SMEs [13, 14]. Additional areas for consideration were invited from attending SMEs through anonymous submission and voting conducted when discussion concluded. Visual resources to support translation to practise were considered as part of this consensus process and considered for ratification by the SMEs present.

The results of the systematic scoping review, the EXIT project research, and the consensus day were made available to the steering group who were also invited to identify and share additional literature/resources for consideration. Evidence was assessed and graded according to FPHC criteria, considering study design, quality, consistency, and directness of evidence.Through discussion and an iterative writing process consensus guidance was drafted.

Draft guidance was submitted and subsequently ratified by the Clinical Standards Group and Executive Committee of the FPHC.

## Result

Forty-three SMEs (Table 1) considered 12 statements (Table 2). All of the statements considered achieved consensus.

**Table 2:** Statements considered and SME voting

Statement	Yes (%)	No (%)	Opt Out (%)
Is self-extrication appropriate if the casualty is experiencing neck or back pain	92	3	5
Is self-extrication appropriate if there are soft neurological signs (e.g. non-dermatomal tingling)	92	0	8
Is self-extrication appropriate if central cord signs?	74	9	17
Actions if hard neurological signs present on initial assessment (e.g. patient unable to move legs) (1) Aim for rapid extrication with gentle patient handling (not absolute movement minimisation)	92	0	8
Actions if neurological signs evolve during self-extrication: (1) Provide immediate support/assisted self-extrication (2) Continue with self-extrication if possible (3) If not possible: Aim for rapid extrication with gentle patient handling (not absolute movement minimisation)	100	0	0
Empowerment of FRS personnel to risk stratify and deliver self-extrication: (1) FRS personnel should be enabled (with appropriate training and governance structures) to deliver self-extrication and assisted self-extrication across all patients. FRS should ensure that this assessment and delivery skill-set is widely available to their patients (2) The U-STEP OUT algorithm can be used by all FRS personnel	97	0	3
Empowerment of lay persons on scene to deliver self-extrication and define limits of this practice (1) The U-STEP OUT algorithm in various forms (app/visual prompt/telephone guided) can be used by lay members of the public and other responding professional groups (e.g. police) following further translational work	100	0	0
Communication on scene/development of shared language/tools. A standardised, national, multi-professional communication tool should be developed, disseminated and appropriate training and oversight provided to ensure adoption into practice	100	0	0
Location of patients post-extrication (1) All patients should be moved to an environmentally safe location (e.g. away from an active highway/under appropriate cover) (2) Patients who self-declare as uninjured or minor injuries and able to meet their own needs should be identified as not requiring further clinical assessment and their details passed to NHS Ambulance service control centre (3) Communication between FRS and clinical response prior to arrival should occur and look to: (A) Optimise patient outcome/experience (B) Optimise the use and availability of clinical and operational resource	100	0	0
Training: The U-STEP OUT algorithm in various forms (app/visual prompt/telephone guided) can be used by lay members of the public and other responding professional groups (e.g. police) following further translational work A multi-disciplinary training package should be developed and made available which empowers clinicians and FRS to deliver self-extrication and assisted self-extrication	97	0	3
The U-STEP OUT tool could be applied to a person of any age who is able to understand	100	0	0
Ratification of Figure: Extrication Decision Tool	94	0	6

**Fig. 2:**
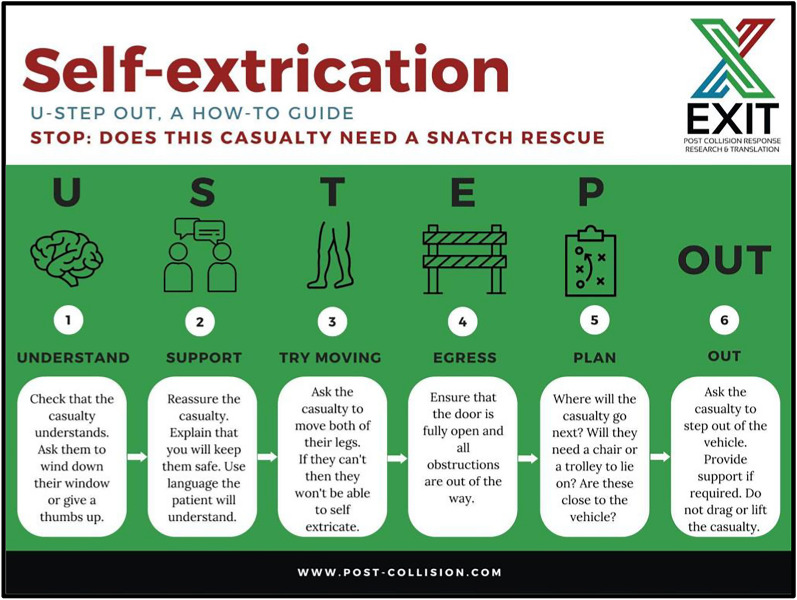
U-STEP OUT

*U-STEP OUT (Fig. 2) Extrication Decision tool (Supplementary Fig. 1)

A total of 14 statements were developed by the steering group and incorporated into the FPHC statement (Table 3). All statements were ratified through the clinical and executive committee.

**Table 3:** FPHC consensus statements

Statement	Level of evidence [15]	Key references
All patients with injury should be considered time dependent. Operational and clinical team members should work together to rapidly develop a bespoke patient centred extrication plan with the primary focus of minimising entrapment time	[IV D]	[4, 5, 10, 16, 17]C
Non-clinicians should be empowered to decide on the extrication mode and deliver this before the arrival of the clinical team	[IV D]	[18] C
Self-extrication or minimally assisted extrication should be the standard ‘first line’ extrication for all patients who do not have contraindications	[III D]	[5, 7, 10, 16] C
Self-extrication decision making for non-clinicians should use an appropriate tool, such as U-STEP OUT	[IV D]	C
Patients who cannot independently self-extricate may benefit from assisted self-extrication	[IV D]	[8] C
In fully conscious patients who do not have neurology it is not necessary to provide manual inline stabilisation in the vehicle	[IV D]	[19, 20] C
If hard neurological signs are present on initial assessment the patient should have a rapid extrication with gentle patient handling	[IV D]	[4] C
Collars reduce neck movement. They should be applied prior to extrication when indicated and removal considered when the extrication phase is complete	[III D]	[11, 20] C
Vehicle relocation, including vehicles in which patients are trapped should be implemented if this will reduce entrapment time	[IV D]	[2] C
Rescuers should be aware that clinical observations may prolong entrapment time and as such should be kept to a minimum	[IV D]	[17, 21] C
Clinical care during entrapment should be limited to necessary critical interventions to expedite safe extrication	[IV D]	[17, 21] C
If a pelvic binder is indicated this should be applied after the process of extrication is complete	[IV D]	S
The psychological impact of extrication should be considered and support mechanisms implemented	[III D]	[6, 7] S
FRS services /brigades and ambulance trusts should ensure regular joint multidisciplinary learning, sharing and case review opportunities	[IV D]	C

C = Derived from consensus day, S = Derived from steering group

## Discussion

Consensus was achieved across a range of extrication related subject areas including: approach and targets, self-extrication, clinical care, immobilisation and delivering a patient-centred rescue. This statement offers a clear sense of direction and supports the ongoing paradigm shift away from absolute movement minimisation centred rescue to a patient and time focused evidence-based approach.

One of the key strengths of this study is it's foundation on a systematic scoping review and learning from the translation of findings from the EXIT project, which has driven significant change in extrication practices. The consensus approach, involving a wide range of stakeholders from prehospital emergency medicine, emergency medicine, and FRS and the inclusion of multiple SMEs representing various regions and organisations, strengthens the validity of the recommendations by incorporating diverse perspectives.

The limitations of the study lie in the inherent constraints of the data sources, particularly relying on retrospective reviews and the lack of randomised controlled trials in this subject area, which historically is typical for prehospital emergency care and areas of complexity such as extrication practice. The study focuses heavily on UK-based stakeholders, which may limit the generalisability of the findings to other international contexts where extrication practices and road traffic injury may differ. Additionally, while the consensus day achieved strong concordance, and the anonymised voting approach offered individual autonomy, it may have limited open debate: a more formal methodology such as a Delphi process could be considered for future FPHC consensus processes.

Compared to previous guidance and (historically) accepted practice in this area, this statement robustly challenges the long-held paradigm of ‘absolute movement minimisation’. This interpretation of the biophysical evidence, which supports self-extrication and use of the quickest extrication technique with appropriate gentle patient handling, represents a significant shift in extrication approach. The incorporation of the U-STEP OUT algorithm, which allows for self-extrication in many cases, empowers not only clinicians but also, subject to appropriate training, firefighters and other non-clinical responders to make decisions that reduce entrapment time and support optimal patient outcomes.

For policymakers, this statement promotes the need to support interdisciplinary training and collaboration between emergency services and complementary guidance in operational clinical practice. Ensuring that rescue personnel and prehospital clinicians work in unison, with shared language and decision-making tools, could lead to significant improvements in both survival rates and the quality of patient care. Policy-makers will need to consider what impact this statement has on enabling bystander and non-clinician led care for the trapped patient.

Despite the advancements provided by this consensus statement, there remain unanswered questions. One key area for future research is improving our understanding of the psychological impact of entrapment and extrication on patients, which, while acknowledged in the guidance, has not been extensively studied. Understanding how prolonged entrapment or self-extrication affects long-term recovery will provide a more holistic view of patient recovery.

Further consideration and evaluation is needed of the broader application of the U-STEP OUT algorithm and similar tools across a range of settings, especially internationally, including low and middle income countries where training levels, resources, and rescue techniques may differ. Additional work is needed in the approach to the physically trapped patient, the limits of self/assisted extrication and the role of non-clinical, non-FRS responders and bystanders in the early care of patients injured in MVC.

Additional research into the specific physiological mechanisms involved in handling during extrication, such as the impact on non-spinal injuries, will also be crucial in refining techniques. The role of technology, such as AI derived scene-specific guidance, could be explored to optimise patient-centred extrication further. We actively encourage the formation of multi-disciplinary/multi-professional data sets for audit and research purposes.

## Conclusion

This consensus statement supports the delivery of an evidence-based, patient-focused extrication. Where evidence gaps exist, we provide balanced, clear and pragmatic solutions. By shifting focus from absolute movement minimisation to a more holistic, time-sensitive, and patient-centred approach, this statement encourages clinicians and policymakers to rethink existing and historical dogmas in extrication. Future research will be essential to build on its findings and address ongoing gaps in the physical and psychological aspects of post-collision care.

## Supplementary Information

Additional file 1.

## Data Availability

No datasets were generated or analysed during the current study.
