# Supplementary material for: Extrication following a motor vehicle collision: a consensus statement on behalf of The Faculty of Pre-hospital Care, Royal College of Surgeons of Edinburgh
Source: Scand J Trauma Resusc Emerg Med. 2025 Jan 6;33:3. doi: 10.1186/s13049-024-01312-z (PMC11706170; doi:10.1186/s13049-024-01312-z)
Supplement: Supplementary file 1 — Additional file 1. [file 13049_2024_1312_MOESM1_ESM.docx]

**Supplementary materials: Extrication following a motor vehicle collision: a consensus statement on behalf of The Faculty of Pre-hospital Care, Royal College of Surgeons of Edinburgh**

This supplementary materials contains:

i) Supplementary figure

ii) Scoping review

iii) Introduction to the EXIT Project

iv) Material circulated to SMEs prior to consensus day

Supplementary Figure 1: Extrication Decision Tool


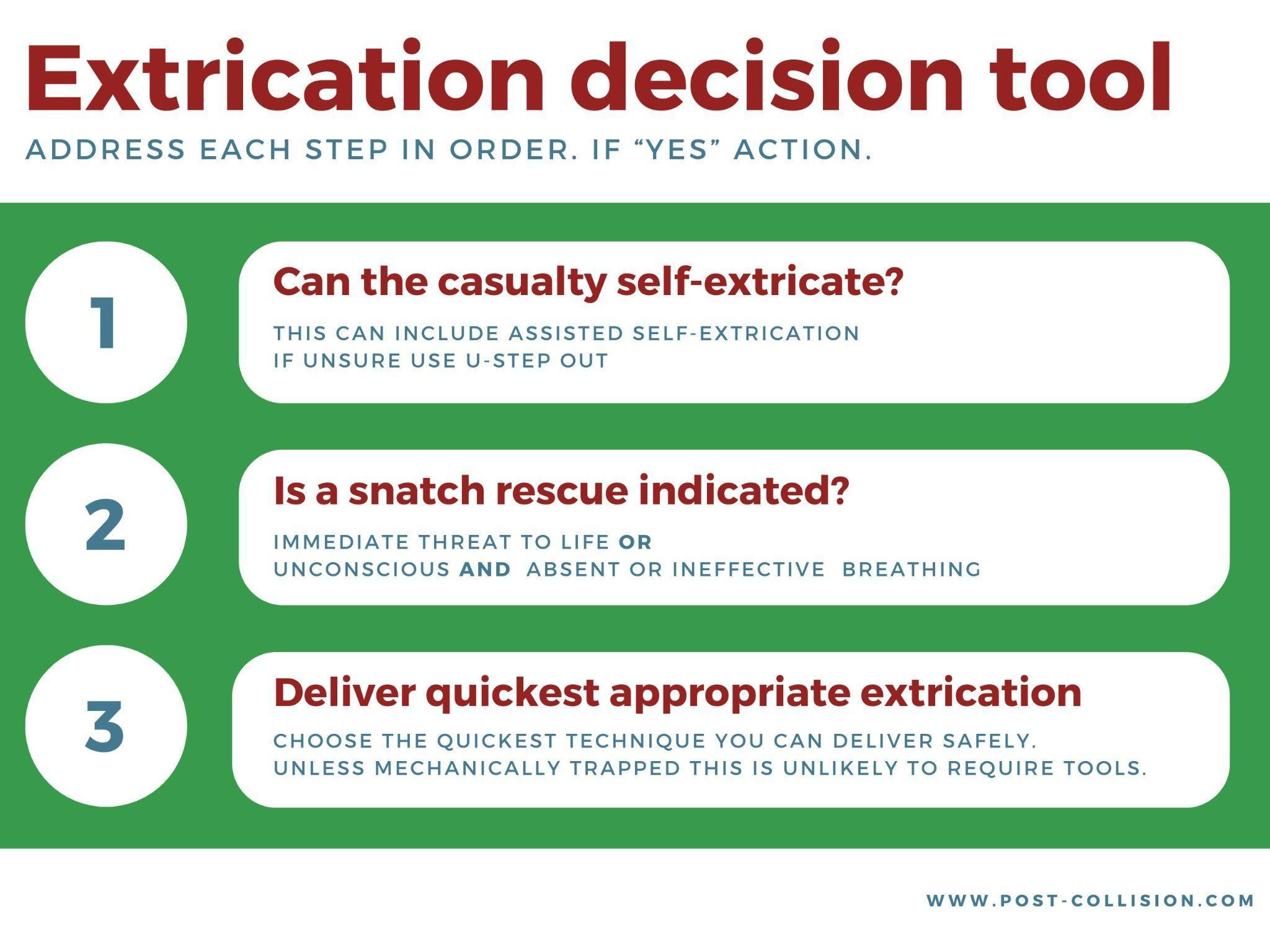


# **Supplementary Text 1: Extrication of patients trapped following a motor vehicle collision: a systematic scoping review of the literature**

**Abstract:**

**Background:** Extrication is the process of removing injured or potentially injured people from their vehicles. The origin of current extrication techniques and paradigms is largely unknown. An understanding of the historical evidence related to motor vehicle collisions (MVCs), injuries and deaths will provide context for accepted, contemporary, extrication practices.

**Methods:** Extrication related search terms were developed and applied across of range of sources including Clinical and health care data, Trial registries, Grey literature, Academic and specific Transport related sources.

**Results:** 7089 articles were identified, following review, 170 are included in this qualitative synthesis. Key themes / categories included: Extrication training and principles, Injures, Immobilisation, Care during entrapment, Clinical response type, Vehicle deformity intrusion entrapment, and Extrication.

**Conclusion:** There is a paucity of published evidence to support the current approach to extrication of entrapped patients following an MVC. Focused studies identifying in detail the injuries and their sequelae associated with entrapment, the biomechanics of current techniques and ensuring that the patient perspective is captured will enable the development of much needed evidence based multidisciplinary guidance.

#

# **Background**

Extrication is the process by which injured, or potentially injured casualties are removed from their vehicles following a motor vehicle collision (MVC) [1]. The origin of current extrication techniques and paradigms is largely unknown. An understanding of the historical evidence related to MVCs, injuries and deaths will provide context for accepted, contemporary, extrication practices.

The review objectives can be defined by the following research questions [2]:

- What is the (historical and scientific) context for current extrication approaches as delivered by rescue services?

- What injuries are sustained by patients who are trapped in their motor vehicles and how does this influence extrication practice?

- What are the needs of patients who are trapped following an MVC, how are these met and following extrication where is their care best delivered?

Extrication is a multidisciplinary undertaking; the literature originates from a wide range of disciplines (clinical, rescue, vehicle design and testing). A systematic scoping approach was considered most appropriate for this review due to both the predicted heterogeneity of the literature and the overarching purpose of this review: to identify gaps in the literature which will aid in the planning of future research [3]. This review will describe and give context to the evolution of the current operational and clinical approach to extrication and identify areas where additional knowledge should be prioritised.

For the purposes of this review, extrication is considered as “the rescue and removal of patients from motor vehicles following a collision”. This review does not include other specialist areas such as rescues from water, caves or collapsed buildings. This review excludes the technical detail of rescue practice and the details of specific rescue equipment. This scoping review is reported to Preferred Reporting Items for Systematic reviews and Meta-Analyses extension for Scoping Reviews (PRISMA-ScR) guidance [4].

# **Methods**

*Search strategy*

This is a scoping review; papers and sources were identified through a systematic search strategy based upon PRISMA methodology. The aim was not to identify a single three-part question – but to identify literature that would inform a deep understanding of extrication and associated themes (see question statements above).

*Development of search terms*

The search strategy was developed with professional librarian assistance, trialled, and further refined to ensure that appropriate references and sources were not missed. The final search strategy is summarised in the box below.

***Box 1.1 Search Terms used***

***
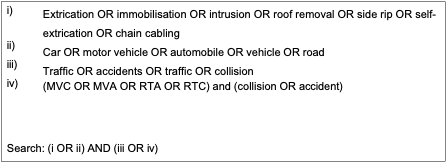
***

The following were searched in August 2021:

Clinical and health care data sources: National Health Services (NHS) available databases using the Healthcare Databases Advanced Search function which includes Medline, EMBASE, CINAHL, EMCare, Healthcare Management Information Consortium (HMIC). From the Cochrane Library we searched the Cochrane Database of Systematic Reviews, Cochrane Central Register of Controlled Trials and Cochrane Clinical Answers. In addition, we searched the Web of Science, Scopus, Health Foundation, Nuffield Trust, PLOS ONE, TRIP, and the Knowledge for Health Care databases.

Trial registries: Clinictrials.gov, WHO International Clinical Trials Registry Platform, EU clinical trials register and the International Standard Randomised Controlled Trial Number ISRCTN registry.

Grey literature sources: The National Grey Literature Collection via the MEDNAR interface, The OAIster^®^ database, The CORE repository, Open Grey, Grey Matters.

Academic sources: E-theses online service (EThOs) from the British Library, Networked Digital Library of Theses and Dissertation (NDLTD), Open Access Theses and Dissertations (OATD)

Other data sources: safetylit.org, the international transport forum web interface, the national academic of science engineering and medicine and the international research council on biomechanics of injury.

*Selection of studies*

Following the search, the Endnote interface (EndNote X9.3.3, Clarivate, Philadelphia, PA, 2013) was used to identify and remove duplicate articles. Sources were included for further review which were and available in the English language and available online or through library services. The remaining studies were reviewed using their abstract and studies which were not relevant to the research questions excluded. A full-text review allowed further exclusion of articles that were not relevant to the research question. Remaining articles were included and their reference list reviewed to identify further articles for inclusion.

*Synthesis*

Following exclusions, full text sources were reviewed with reference to the research questions and a broad analysis of the domains identified conducted; articles were grouped into domains, reviewed and included in the narrative discussion.

# **Results**

An initial total of 16,413 documents were identified through the search strategy. This was reduced to 7089 following removal of duplicates. One hundred and seventy papers were identified that were relevant to the research questions. Results are summarised in Figures 1.1 and 1.2.

**Figure 1.1: Studies screened and included (adapted from PRISMA)**


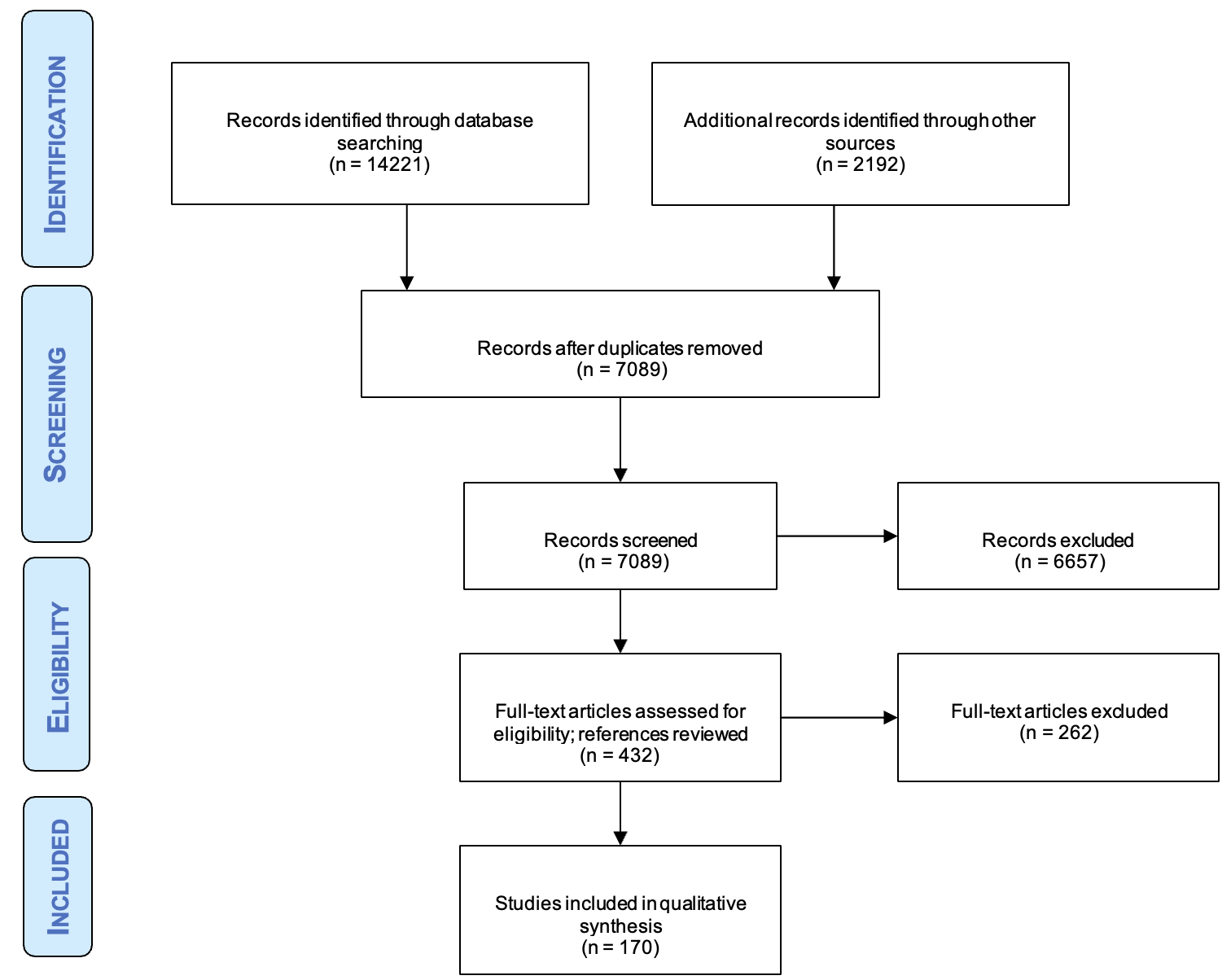


Figure 1.2 Outlines the domains which were identified by full-text review. These are: extrication training and principles, injuries associated with MVC and extrication, immobilisation, care during entrapment, clinical response type and vehicle deformity, intrusion, entrapment and extrication time, other related papers and extrication specific papers.

**Figure 1.2 Domains identified**

**
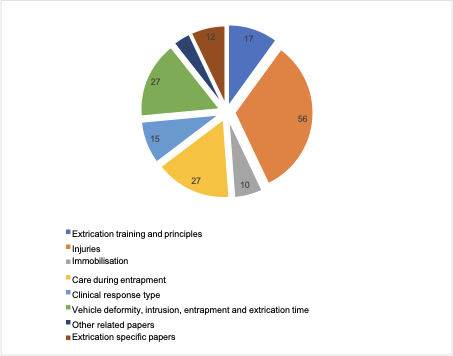
**

# **Discussion**

The scoping review identified studies from a wide range of disciplines and backgrounds. The study types were diverse including computer modelling of accidents and energy transfer, retrospective chart review and database review studies, individual case reports, case series, post-mortem studies, biomechanics, kinematics and mannequin-based studies. There were no randomised controlled trials, no interventional studies of clinical or operational care, and no prospective cohort studies. There were only two unique prospective ‘real world’ extrication focused analyses [5,6].

Common domains in the literature are explored in the following sections.

*Extrication training and principles*

The principle of movement minimisation is a key paradigm of contemporary extrication practice [1]. The earliest papers that discussed the priorities to achieve in extrication are from medical journals in the 60’s and 70’s. These papers identify that patients may have time dependent injuries and state the importance of movement minimisation to prevent avoidable secondary spinal injury following an MVC [7–13]. The assertions in relation to movement minimisation are made without reference to specific cases, case series or published data. The primacy of movement minimisation during the process of extrication emerges in extrication manuals and guidance aimed at rescue services from the 1970’s and onwards [14–22]. The manuals and textbooks were unreferenced in respect to the origin of, or justification for the primary focus on spinal injuries above other injuries in the development of extrication paradigms.

*Injuries*

Early post-mortem studies identify the wide range of injuries from which patients injured in MVC succumbed [23]. Even in these early studies (and before the introduction of modern safety systems) the rate of spinal cord injury and particularly isolated spinal injury (which might justify movement minimisation extrication methods for extrication) was low compared to other injuries; 0.8% of fatalities had a spinal cord injury and 70% had a head injury [23]. With the adoption of seatbelts, the primary injuries and death caused by ejection were minimised with an associated drop in mortality, but new injuries originated: facial fractures from impacting with the internal surfaces of the car and abdominal injuries cause by the belts themselves [24,25].

Much of the literature focuses on injuries in isolation, as opposed to patterns or constellations of injuries. Several papers consider individual cases, case series and mechanism type for a variety of individual injuries including limb [26–29], aortic [30–34], pelvic [35–37] brain [38–41], abdominal [30,42,43] and other body areas and injury types [44–50]. Injured obese patients were identified as having worse outcomes [51].

Spinal injuries

Case reports and retrospective reviews of routinely collected data of severe spinal injury following an MVC featured both adults and children [52–56]. Mezue *et al.* reported failures in prehospital immobilisation and careful handling in patients with subsequently proven spinal cord injury [57]. The authors report that 94.1% of patients in their series were extricated by bystanders and only 36% of the patients had any attempt at immobilisation prior to hospital arrival, the authors report an association between adequate immobilisation and transport and improved function at discharge (p=0.003) [57].

Sochor and colleagues identify scene factors which predict the presence of a clinically important spinal injury [58]. In front seat restrained drivers or passengers between 16 and 60 years of age, if the glass in their car was unbroken following an MVC that the rate of clinically important spinal injury was very low. The sensitivity for the GLASS rule was 95.20% (95% CI 91.45–98.95%), speciﬁcity was 54.27% (95% CI 53.44–55.09%), and the negative predictive

value was 99.92% (95% CI 99.86–99.98%) [58].

Injuries in those who are trapped

Siegel *et al.* compared injuries in patients who required extrication compared to those that did not. They found a higher rate of brain (51% v’s 35%, non-significant), lower extremity (58% v’s 30%, p<0.003) and splenic injuries (22%, v’s 10%, p<0.02) in patients that required extrication compared to those that did not [59].

Sanson *et al.* report a case series of HEMS delivered critical care interventions on patients who were trapped. They report a high injury load including tension pneumothorax (11.8%), major head injury (39%), and non-compressible haemorrhage (34.7%) [60]. Wilmink reports a case series of entrapment MVCs attended by a UK Helicopter Emergency Medical Services (HEMS) [6], with a high injury load (median ISS 17, range 1-59) in entrapped patients and an associated high mortality (10%). They note that in their case series isolated spinal cord injury did not occur with a majority of patients with severe spinal injury having an associated major head injury affecting their level of consciousness and therefore limiting the efficacy of clinical assessment (36% of all patients had a head or spinal injury) [6]. Westhoff *et al.* consider trapped patients from both passenger vehicles and trucks and report a high degree of severe single system injury (68.7% to the head, 23.5% to the neck, 50.8% to the chest, 43.6% to upper extremities, 15.4% to the abdomen, 16.4% to the pelvis, and 52.9% to lower extremities) and multiple injuries in trapped patients [61].

The literature identified in this scoping review does not provide contemporaneous data that allows us to accurately report the rate of spinal cord injury in entrapped patients. We can conclude that the rate of time dependent injury is high in the those who are entrapped but it is unclear if this is leads to poor outcomes or if entrapment alone might lead to increased morbidity and mortality.

*Non-physical injuries*

Non-physical injuries are a frequent cause of long-term morbidity and affect the quality of life of those who suffer from them [62]. MVC’s are associated with a high rate of post-traumatic stress disorder (PTSD) and other psychological sequelae both in children and adults [63–78]. Mayou’s group compared those with multiple injuries following an MVC and those with whiplash injuries alone, they report that in the acute phase (within one month) following the accident that those with multiple injuries were more likely to have an acute stress reaction (41%, comparator not reported); interestingly long-term psychological outcomes did not appear to be correlated with severity of injury [65]. Mosaku K *et al*. performed a complimentary study that identified that clinical factors did not predict long term psychological outcomes [77]. Heron-Delaney conducted a systematic review with the intention of identifying factors that predict PTSD in adult MVC survivors and found that the prevalence of PTSD varied from 6- 45% with a “perceived threat to life” being a significant predictor of long term poor psychological recovery [79]. Watts and team found that up to 77% of post MVC victims admitted to hospital were likely to have an “acute psychiatric disorder” with 11-15% seeking or receiving professional counselling [69].

Arnberg and team considered the long-term PTSD outcomes of children following an MVC; they found a high prevalence of stress reactions at nine months following the event (50-69%), with PTSD symptoms still present in 18% of their sample at 20 years [73].

A single paper considered the experience of patients that were trapped (due to spinal cord injury) following an MVC [80]. Sepahvand introduces the concept of “scene shock” in which the injured and untrained bystanders fall into a “state of instability” leading to emotionally driven decisions and subsequent behaviours that lead to desperate, unplanned rescue efforts which may contribute to secondary spinal cord injury [80].

This review confirms that non-physical injuries are common following MVC. Specific data on entrapment or extrication as a risk factor for non-physical injury was not identified. We hypothesise that being trapped would be considered by patients to be a “threat to life” and as such this group may be at higher risk of poor psychological outcomes and long-term symptoms.

Importantly, no data was available that recounted the patient experience of entrapment or extrication or considered if changes to this area of practice may improve the patient experience.

*Immobilisation*

Prehospital services use immobilisation devices to mitigate against movement and ensure or return anatomical normality [81]. Immobilisation can include the application of a femoral traction device, a pelvic sling or the ‘triple immobilisation’ of a cervical collar, head blocks and a long board or scoop stretcher. Two papers in this review reported pelvic immobilisation techniques and suggest that they may be appropriate for use in entrapment [82,83]. A small number of papers reported methods of paediatric immobilisation using novel techniques or adapting standard prehospital equipment [84–86].

Recent publications challenge the ubiquitous application of cervical collars or the use of spinal boards in the extrication and transportation phase following an MVC [87–90]. These papers, based on expert opinion and an analysis of ‘excess imaging’ associated with immobilisation suggest alternative approaches including gentle patient handling techniques and self- extrication [87–90].

Immobilisation, particularly the use of cervical collars has been a subject of increasing enquiry and consideration over the last 15 years [91–93]. Authors have challenged the harm / benefit of collar application, particularly in conscious trauma patients [93]. The use of such immobilisation devices specifically in the context of entrapment and extrication is discussed later in this review.

*Care during entrapment*

Papers were identified that related to the delivery of patient care, minimisation of patient harm or improvement of patient experience during entrapment. No papers were identified which included any description of patient experience or collection of patient generated data (e.g. pain scores).

Single case studies were presented which identify pain and the potential for hypothermia as issues that benefit from mitigation whilst the patient remains trapped [94–97]. A series of four cases supported by a literature review identify that ketamine is well suited for meeting the analgesic needs of a trapped patient [98]. Further papers presented general principles and opinion on pain management options [99,100].

A surprisingly large number of mannequin-based studies evaluated the use of a wide variety of laryngoscopes or supraglottic airway devices for the placement of endotracheal tubes in

entrapped mannequins in various positions [101–115]. Individual case studies and small case series supported the use of supraglottic airway devices in extremis [116–118]. A single retrospective chart review of airway management published as an abstract recognised the challenges of intubation in the entrapped patient [119].

The literature in this area is limited to a single case series, expert opinion and mannequin studies looking exclusively at airway management. Literature was not identified that defined patient’s clinical needs and priorities for the management during the entrapment and extrication phase.

*Clinical response type*

The utility of bystanders at the scene of an MVC was considered by several authors. Thierbach *et al.* identified that bystanders were more likely to help with those with moderate injuries than patients with severe injuries and advocated for more advanced widely available bystander training [120]. Heightman and Bhalla discuss the potential utility of bystander care to reduce mortality and morbidity, especially with those with specific training, kit and authorisation [121,122]. Bhalla reflects on the potential medico-legal culpability for bystanders in providing immediate care and how this might be overcome by training and authorisation to act [121,122].

Two studies from the 1990’s identified that entrapment was associated with severe injuries, and this resulted in complex patient care needs which were often unmet [123,124]. Many papers advocated for physician attendance at scene for entrapment trauma [125–131]. A single prospective cohort study considering all mechanisms of major trauma found no survival benefit when a physician was present (OR of 1.16 (95% confidence interval = 0.97 to 1.40, p

= 0.11).

Byrne *et al.* report that longer response times were associated with higher rates of mortality [132], whilst Gauss and team noted the association between prolonged prehospital time and poor patient outcomes [133].

Patients who are trapped have on average longer prehospital timelines and as such may have an excess mortality for this reason alone [134]. The benefits and potential harms of bystanders to patients and the ideal clinical response model cannot be surmised from the literature available to this review.

*Vehicle deformity, intrusion, entrapment and extrication time*

These papers considered patient and incident-based factors which predicted (or failed to predict) mortality, injury or the need for trauma centre care. The papers offered different

perspectives as to the utility of incident-based factors both in isolation and combined with injury, physiological or patient demographic factors.

The factors of interest to this review are the association between vehicle structural deformity (external), intrusion into the passenger compartment and the requirement for the extrication of a patient. These factors are important to our question of the inter-relation of patient injury and their ability to self-extricate.

Three papers considered the accuracy of the data recorded by both paramedics and emergency physicians in terms of scene characteristics (such as need for extrication). Poor completion of prehospital records and poor correlation between findings at scene and subsequent analysis led to both under and over triage (EMS record accuracy median 28.5%, range 0-100%) [135–137].

Deformity

External vehicle deformity was found to be important when combined with intrusion in the absence of air bags (OR 5.2, 95% CI 2.525–10.780) [138]. Deformity was also important in predicting mortality in older patients (differences in mortality were associated with age (OR 6.92,95% CI 1.2-38.9) and a high vehicle deformity (OR 3.28, 95%C1 1.5-6.8)[139].

Intrusion:

Studies reached different conclusions when considering intrusion alone as a predictor of injury, mortality or trauma system utilisation [140,141]. One paper identified supported the utilisation of intrusion alone in frontal collisions as an indicator of major trauma and as such should feature on major trauma triage tools [142]. A paper from 1996 reported the utility of intrusion of >24 inches as a triage criterion but found other mechanistic criteria were not useful [143]. Davidson *et al.* found that intrusion of more than 12 inches were useful in predicting trauma centre utilisation over and above physiological criteria; they found mechanistic criteria particularly useful in older patients without physiological derangement. Intrusion of greater than 12 inches had a PPV of 10.4% (95% CI, 9.5-11.3) to predict severe injury; steering wheel collapse had a PPV of 25.7% (95% CI, 23.0-28.4%) for the same outcome [144].

More recent reviews did not support intrusion as a stand-alone predictor of injury, and instead suggest that patients triaged on intrusion alone had low Injury Severity Score (ISS) and a high discharge rate (ISS was 5 (1.75, 10.25) and 39.5% were discharged from the Emergency Department (ED)) [145,146]. Simon *et al.* recommend that if certain mechanistic features were present and no evidence of physiological disturbance then an initial clinical review of the patient should occur and then upgrade to a trauma team if required [147]

The combination of intrusion and entrapment, which are often inter-related, was identified as useful for predicting patient mortality. When adjusted for age and sex, the following mechanism of injury (MOI) were associated with mortality: passenger space intrusion (OR 1.74; CI 1.18, 2.57), extrication (OR 2.16, CI 1.14, 4.04), ejection (OR 8.33; CI 4.68, 14.83)

and occupant fatality (OR 2.28; CI 0.50, 10.40) [148]. Entrapment and extrication

Many groups identified that entrapment, particularly when associated with prolonged or difficult extrication (typically defined as > 20 minutes) was a useful predictor of injury (multivariate OR 2.5, 1.1–6.0, p=0.04), and was a more sensitive and specific criterion for trauma centre utilisation than other mechanistic features [149–156].

This finding was not universal with two authors recommending that the need for extrication in isolation should be removed from triage guidance as it led to considerable over-triage [157,158].

There were no studies concerning vehicle deformity or extrication which included children. However, intrusion was found to be associated with increased injury in children, with a direct relationship between the amount of intrusion and associated injuries (4.0% increase in AIS3+ injuries for each cm of intrusion (95% CI = 2.7-5.2%) [159–161].

Other related papers

Ryb *et al.* suggested that patient mobility post collision was more useful than mechanistic factors in triaging patients to an appropriate facility; self-extrication under-triaged by 0.4% as a predictor of death[162]. Schulman and colleagues developed a composite “Scenescore” consisting of weighted values for age, collision type, impact location, airbag deployment, steering wheel deformity, intrusion, and restraint use; they suggest a score of 8 offers optimal performance (sensitivity 76%, specificity 46%) to assist with triage decisions [163]. Technological solutions were also suggested utilising automatic crash notification or vehicle telemetry to predict injuries and inform response [164–166].

As might be expected the conclusions and recommendations varied with the era of analysis and publication. This may be in part to the increased safety of vehicle systems, the development of vehicles in terms of crumple zones, changes in the way patients were considered trapped or needed extrication and the individual capability and acceptable over- triage rates of the system under consideration.

*Extrication specific papers:*

Nutbeam *et al.* prospectively collected data at the scene of entrapment MVC, then used this to report factors that predict the need for extrication, the factors which affect this time and the number of extrications in which physical or actual entrapment occurs (10%) [5,134,167]. This low rate of physical entrapment (10% of all extrications), the time taken for extrication (median 30 minutes) and the increased mortality seen with both entrapment and increasing time between injury and arrival at hospital demonstrates the importance of the entrapped patient as an area where increased knowledge and decreasing the rate and time of entrapment may lead to improved patient outcomes.

There were very few papers that considered the effect of extrication technique on entrapment time or patient outcomes. Lars and Fattah both demonstrate the speed of chain cabling type techniques which are used in Scandinavian countries but not frequently used elsewhere compared to more traditional techniques in experimental conditions [168,169].

There are a number of papers that report bio-mechanical analysis using various methodologies and a range of extrication types. Bucher *et al.* found that utilising a KED (Kendrick extrication device) resulted in less spinal movement in patients with a normal body mass index (BMI) but increased spinal movement in obese patients [170]. Shafer *et al.* performed a pilot study which concluded that allowing an individual to exit a car under their own volition (self-extrication) with a cervical collar in place may result in the least amount of motion compared to exiting with paramedic assistance [171]. These findings were reinforced by Engsverg, Gabrieli, Haske and Dixon and their respective teams across a number of extrication methods using a variety of biomechanical methods and outcome measures [172– 176].

*Where are the gaps?*

Considering the large number of patients whose clinical care, timeline to hospital and patient experience may have been adversely affected by their trapped status, there is little focused literature which allows an understanding of key areas of this phenomenon which would enable an EBM approach to the development of evidence-based extrication guidance.

**Figure 1.3 The EBM Triad**

**
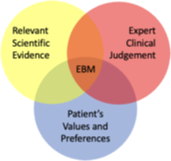
**

Areas of ‘relevant scientific evidence’ where data is not available or not sufficient includes the difference in injury patterns between trapped and not trapped patients, the difference in outcome between trapped and not trapped patients, the efficacy of extrication techniques to minimise movement and their clinical or outcome implications. There is not currently evidence that enables us to understand ‘patient values and preferences’; we do not have data which supports an understanding of the patient experience of extrication and how this may be improved. Despite a large number of case reports and papers from single or small groups of experts there is no coherent, consensus “expert clinical judgement’ which bridges the rescuer- clinician divide in the current literature. The absence of multidisciplinary guidance based on the best available evidence demonstrates another notable gap in relation to this important patient group.

Our understanding of these important areas of research could be improved by targeted studies analysing high-quality data sources which allow comparison of injuries, injury patterns and outcomes between trapped and not-trapped patients following an MVC. Such analyses will be enhanced by reporting the frequency of isolated spinal injuries that may be exacerbated by movement and time-critical injuries such as significant head injuries. These analyses will contextualise the risk of secondary spinal injury, the risks of patient deterioration whilst trapped and help us to understand the potential for self-extrication. Sub-analyses which allow comparisons between patients of different ages, sex and body habitus will further inform decision making in this area.

Current biomechanical data of extrications are limited to small numbers of extrications across a small pool of healthy volunteers. Where possible, real-world data should be collected to inform our understanding of the performance of currently deployed extrication techniques. If

real-world data collection is not possible then researchers should deliver adequately powered studies which consider all extrication types across a range of people.

Good evidence-based medicine requires the consideration of patient values and preferences [177]. The absence of the patient voice from the current evidence base is notable and rectifying this should be a target for future research. Patient surveys and interviews will assist in capturing the patient perspective and routinely collected data in this area should include patient experience. Patient priorities should be identified, and patient representatives should be engaged in the development of guidance for the care of patients whilst they are entrapped and subsequently extricated.

Solutions for the evidence gaps identified above will enable the development of much needed evidence-based multidisciplinary guidance through consensus processes.

*Limitations of this scoping review*

We aimed for a comprehensive search strategy; however, it may have missed studies that were important to our defined questions. Steps were taken to keep the inclusion criteria broad and included a large number of grey literature sources; which in turn required the review of a large number of papers. By defining questions in advance, we attempted to produce a decision-making process which was predictable and reproducible, but this was not confirmed through any verification process. A single researcher applied the questions and made decisions regarding inclusion and exclusion criteria, which may have improved the reliability of these decisions but threatens the reproducibility if repeated by another person or team.

The nature of the scoping review does not require a formal risk of bias assessment. The broad nature of the review does not allow for the comprehensive synthesis of all domains, nor does it provide the specificity to identify immediate recommendations to improve extrication practice.

#

# **Conclusions**

There is a paucity of published evidence to support the current approach to extrication of entrapped patients following an MVC. Focused studies identifying in detail the injures and their sequelae associated with entrapment, the biomechanics of current techniques and ensuring that the patient perspective is captured will enable the development of much needed evidence based multidisciplinary guidance.

References:

1. Dunbar I. Vehicle Extrication – The Next Generation. LUKAS; 2021.

2. Peters MDJ, Godfrey CM, Khalil H, McInerney P, Parker D, Soares CB. Guidance for conducting systematic scoping reviews. Int J Evidence-based Healthc. 2015;13:141–6.

3. Munn Z, Peters MDJ, Stern C, Tufanaru C, McArthur A, Aromataris E. Systematic review or scoping review? Guidance for authors when choosing between a systematic or scoping review approach. Bmc Med Res Methodol. 2018;18:143.

4. Tricco AC, Lillie E, Zarin W, O’Brien KK, Colquhoun H, Levac D, et al. PRISMA Extension for Scoping Reviews (PRISMA-ScR): Checklist and Explanation. Ann Intern Med. 2018;169:467–73.

5. Nutbeam T, Fenwick R, Hobson C, Holland V, Michael P. The stages of extrication: a prospective study. Emerg Med J. 9AD;31:1006–8.

6. Wilmink ABM, Samra GS, Watson LM, Wilson AW. Vehicle entrapment rescue and pre-hospital trauma care. Inj. 1996;27:21–5.

7. Hall MH. The Trapped Motorist. Lancet (London, England). 1965;1:904–6.

8. Farrington JD. Extrication of victims—surgical principles. Journal of Trauma - Injury, Infection and Critical Care. 1968;8:493–512.

9. Kossuth LC. The extrication of victims from the accident. Arizona medicine. 1969;26:128–30.

10. Snook R. Extrication of trapped casualties. British medical journal. 11AD;4:478–80.

11. Snook R. Medical aid at road accidents: extrication. Update. 1973;6:1625–40.

12. Kimball KF. Principles and concepts of extrication. The Nebraska medical journal. 1971;56:465–6.

13. Mahon AGM. Medical management of the trapped patient. South African Medical Journal. 1977;52:683–5.

14. Florence DW. Extrication -- rescue techniques. Emergency medical services. 1976;5.

15. Kidd JS, Czajkowski JD. Commanding the extrication scene. JEMS: Journal of Emergency Medical Services. 1987;12:30–3.

16. Czajkowski J, Kidd S. Extrication challenges. 10 tips for EMS crews working at extrication scenes. EMS on scene. JEMS. 2001;26:50–61.

17. Schollinski L, Grill C, Donner E, Nerlich M. The “Entrapped” trauma of vehicle occupants as seen by the accident expert. “Audi” traffic accident research project AARU. Der Notarzt. 2001;17:63–6.

18. Kanz KG, Schmoller G, Enhuber K, Holzl G, Sturm JA, Mutschler W. Algorithm for extrication and medical care in vehicular trauma. Unfallchirurg. 2002;105:1015–21.

19. Calland V. Extrication of the seriously injured road crash victim. Emergency Medicine Journal. 2005;817–21.

20. Baker L. Enhancing Vehicle Extrication Instruction. Fire engineering. 2006;159:163–8.

21. Inspectorate Great Britain. Fire Service. Fire and rescue service manual. London: TSO; 2007.

22. Politis J, Dailey M. Extrication Fundamentals Proper care of the entrapped patient. JEMS. 2010;35:41–7.

23. Sevitt S. Fatal road accidents. Injuries, complications, and causes of death in 250 subjects. Brit J Surg. 1968;55:481–505.

24. Huelke DF, Chewning WA. Comparison of Occupant Injuries With and Without Seat Belts. SE-Paper 690244. 1969;

25. O’Day J, Scott RE. Safety belt use, ejection and entrapment. Health education quarterly. 1984;11:141–6.

26. Crandall JR, Martin PG, Sieveka EM, Pilkey WD, Dischinger PC, Burgess AR, et al. Lower limb response and injury in frontal crashes. Accident Analysis and Prevention. 1998;30:667–77.

27. Howard A, Rothman L, McKeag AM, Pazmino-Canizares J, Monk B, Comeau JL, et al. Children in side-impact motor vehicle crashes: Seating positions and injury mechanisms. Journal of Trauma - Injury, Infection and Critical Care. 2004;56:1276–85.

28. Ye X, Poplin G, Bose D, Forbes A, Hurwitz S, Shaw G, et al. Analysis of crash parameters and driver characteristics associated with lower limb injury. Accident Analysis and Prevention. 2015;83:37–46.

29. Laituri TR, Henry S, Sullivan K, Prasad P. Lower-body injury rates in full-engagement frontal impacts: Field data and logistic models. SAE Technical Papers. 2006;

30. Reiff DA, Jr GM, Windham ST, Rue ILW, Metzger J, Doss M. Identifying injuries and motor vehicle collision characteristics that together are suggestive of diaphragmatic rupture. Journal of Trauma - Injury, Infection and Critical Care. 2002;53:1139–45.

31. Sciences KB th ASU of A at BBALC for I, gerald.mcgwin@ccc.uab.edu, Metzger J, Moran SG, W. IR L. Occupant- and collision-related risk factors for blunt thoracic aorta injury...includes discussion. Journal of Trauma. 2003;54:655–62.

32. Carol C, David BH, Eastman AB, Lisa HT, Sharon P, Steve E, et al. Motor vehicle-related cardiac and aortic injuries differ from other thoracic injuries. Journal of Trauma. 5AD;62:1462–7.

33. Siegel JH, Belwadi A, Smith JA, Shah C, Yang K. Analysis of the Mechanism of Lateral Impact Aortic Isthmus Disruption in Real-Life Motor Vehicle Crashes Using a Computer-Based Finite Element Numeric Model: With Simulation of Prevention Strategies. Journal of Trauma-Injury Infection and Critical Care. 2010;68:1375–94.

34. Chang ET, Holcombe S, Kohoyda-Inglis C, MacWilliams JB, Parenteau C, Wang L, et al. Morphomic analysis of thoracic injury risk in live subjects from real world motor vehicle crashes. Journal of Surgical Research. 2014;186:659–60.

35. Moffatt CA, Mitter EL, Martinez R. Pelvic fractures crash vehicle indicators. Accident; analysis and prevention. 1990;22:561–9.

36. Schiff MA, Tencer AF, Mack CD. Risk factors for pelvic fractures in lateral impact motor vehicle crashes. Accident Analysis and Prevention. 2008;40:387–91.

37. Vishal B, Carol C, Jeanne L, Alexandra S, Gail T, Raul C. Is bigger better? The effect of obesity on pelvic fractures after side impact motor vehicle crashes. Journal of Trauma. 9AD;67:709–14.

38. Coimbra R, Conroy C, Hoyt DB, Pacyna S, May M, Erwin S, et al. The influence of damage distribution on serious brain injury in occupants in frontal motor vehicle crashes. Accident Analysis and Prevention. 2008;40:1569–75.

39. Nance ML, Kallan MJ, Arbogast KB, Park MS, Durbin DR, Winston FK. Factors Associated With Clinically Significant Head Injury in Children Involved in Motor Vehicle Crashes. Traffic Injury Prevention. 2010;11:600–5.

40. Parenteau C, Viano DC. Basilar Skull Fractures by Crash Type and Injury Source. SAE International Journal of Passenger Cars - Mechanical Systems. 2011;4:917–28.

41. Viano DC, Parenteau CS. Brainstem injury in motor vehicle crashes. Traffic Inj Prev. 2017;18:730–5.

42. Tso EL, Beaver BL, Haller JA. Abdominal injuries in restrained pediatric passengers. Journal of pediatric surgery. 1993;28:915–9.

43. Klinich KD, Flannagan CAC, Nicholson K, Schneider LW, Rupp JD. Factors Associated with Abdominal Injury in Frontal, Farside, and Nearside Crashes. SAE Technical Papers. 2010;2010-November.

44. Jr GM, Reiff DA, Moran SG, Rue ILW. Incidence and characteristics of motor vehicle collision-related blunt thoracic aortic injury according to age. Journal of Trauma - Injury, Infection and Critical Care. 2002;52:859–65.

45. James KK, Robert K, Jonathan LW, Charles M, Avery BN, Hunter W, et al. Renal injury mechanisms of motor vehicle collisions: analysis of the crash injury research and engineering network data set. Journal of Urology. 8AD;178:935–40; discussion 940.

46. Weaver AA, Gayzik FS, Stitzel JD. Biomechanical analysis of pulmonary contusion in motor vehicle crash victims: A crash injury research and engineering network (CIREN) study. 2009. p. 364–9.

47. Pathak H, Borkar J, Dixit P, Shrigiriwar M. Traumatic asphyxial deaths in car crush: Report of 3 autopsy cases. Forensic science international. 2012;221:e21.

48. Ekambaram K, Frampton R, Lenard J. Factors associated with chest injuries to front seat occupants in frontal impacts. Traffic Injury Prevention. 2019;20:S37–42.

49. Lisa HT, David BH, Eastman AB, Michael JS, Frank K, Tom V, et al. The impact of safety belt use on liver injuries in motor vehicle crashes: the importance of motor vehicle safety systems. Journal of Trauma. 7AD;63:300–6.

50. Viano DC, Parenteau CS. Field Accident Data Analysis of 2nd row children and individual case reviews. Sae Technical Pap Ser. 2008;

51. Bhatti JA, Nathens AB, Redelmeier DA. Driver’s obesity and road crash risks in the United States. Traffic Injury Prevention. 2016;17:604–9.

52. Stawicki SP, Holmes JH, Kallan MJ, Nance ML. Fatal child cervical spine injuries in motor vehicle collisions: Analysis using unique linked national datasets. Injury. 2009;40:864–7.

53. Martínez-Lage JF, Alarcón F, Alfaro R, Gilabert A, Reyes SB, Almagro MJ, et al. Severe spinal cord injury in craniocervical dislocation. Case-based update. Child’s Nervous System. 2013;29:187–94.

54. Lozen AM, Pace J, Yoganandan N, Pintar FA, Cusick JF. Unilateral atlanto-axial fractures in near side impact collisions: An under recognized entity in cervical trauma. Journal of Craniovertebral Junction and Spine. 2014;5:33–7.

55. Murphy RF, Davidson AR, Kelly DM, Warner WC, Sawyer JR. Subaxial Cervical Spine Injuries in Children and Adolescents. Journal of Pediatric Orthopaedics. 2015;35:136–9.

56. Dubrovin IA, Sedykh EP, Mosoyan AS, Bychkov AA, Akhmetova DN. The characteristic features of vertebral lesions in the victims of a road traffic accident with intrusion into the passenger car compartment. Sudebno-Meditsinskaya Ekspertiza. 2018;61:12–5.

57. Mezue WC, Onyia E, Illoabachie IC, Chikani MC, Ohaegbulam SC. Care Related and Transit Neuronal Injuries after Cervical Spine Trauma: State of Care and Practice in Nigeria. Journal of Neurotrauma. 2013;30:1602–7.

58. Sochor M, Althoff S, Bose D, Maio R, Deflorio P. Glass Intact Assures Safe Cervical Spine Protocol. J Emerg Medicine. 2013;44:631-636.e1.

59. Siegel JH, Mason-Gonzalez S, Dischinger PC, Read KM. Causes and Costs of Injuries in Multiple Trauma Patients Requiring Extrication from Motor Vehicle Crashes. The journal of trauma. 1993;35.

60. Sanson G, Bartolomeo SD, Nardi G, Albanese P, Diani A, Raffin L, et al. Road traffic accidents with vehicular entrapment: incidence of major injuries and need for advanced life support. European journal of emergency medicine : official journal of the European Society for Emergency Medicine. 1999;6:285–91.

61. Westhoff J, Haasper C, Otte D, Probst C, Krettek C, Richter M. Motor vehicle accidents with entrapment. A medical and technical investigation of crash mechanism, injury pattern and severity of entrapment of motor vehicle occupants between 1983 and 2003. Der Chir Zeitschrift Für Alle Gebiete Der Operativen Medizen. 2007;78:246–53.

62. Rissanen R, Berg H-Y, Hasselberg M. Quality of life following road traffic injury: A systematic literature review. Accid Analysis Prev. 2017;108:308–20.

63. Sigmon HD. Helping your long-term trauma patient travel the road to recovery. Nursing. 1984;14:58–64.

64. Bueno MM. The effects of appraisal, coping and post-traumatic stress disorder in drivers and passengers injured in motor vehicle crashes. Effects of Appraisal, Coping & Post-traumatic Stress Disorder in Drivers & Passengers Injured in Motor Vehicle Crashes. 1993;207 p.

65. Mayou R, Bryant B, Duthie R. Psychiatric consequences of road traffic accidents. Brit Med J. 1993;307:647.

66. Bryant RA, Harvey AG. Avoidant Coping Style And Posttraumatic Stress Following Motor-Vehicle Accidents. Behaviour Research and Therapy. 1995;33:631–5.

67. Bryant RA, Harvey AG. Initial posttraumatic stress responses following motor vehicle accidents. Journal of Traumatic Stress. 1996;9:223–34.

68. Harvey AG, Bryant RA. The relationship between acute stress disorder and posttraumatic stress disorder: A 2-year prospective evaluation. Journal of Consulting and Clinical Psychology. 1999;67:985–8.

69. Watts R, Horne D, Sandells J, Petrie M. The need for acute hospitals to provide counselling following motor vehicle accidents. Australian health review : a publication of the Australian Hospital Association. 1996;19:93–103.

70. Yutaka M, Daisuke N, Satomi N, Yoshiharu K, Masato H, Yasuhiro O. Incidence and prediction of psychiatric morbidity after a motor vehicle accident in Japan: The Tachikawa Cohort of Motor Vehicle Accident Study. Critical Care Medicine. 10AD;36:74–80.

71. Beck JG, Grant DM, Read JP, Clapp JD, Miller LM, Palyo SA, et al. The Impact of Event Scale-Revised: Psychometric properties in a sample of motor vehicle accident survivors. Journal of Anxiety Disorders. 2008;22:187–98.

72. Wu KKY. A randomised controlled trial of brief cognitive-behavioural therapy and a self-help booklet as early interventions for post-traumatic stress after road trajfic accident. East asian archives of psychiatry. 2010;20:46‐47.

73. Arnberg FK, Rydelius PA, Lundin T. A longitudinal follow-up of posttraumatic stress: From 9 months to 20 years after a major road traffic accident. Child and Adolescent Psychiatry and Mental Health. 2011;5.

74. Bovin MJ, Marx BP. The Importance of the Peritraumatic Experience in Defining Traumatic Stress. Psychological Bulletin. 2011;137:47–67.

75. Suliman S, Troeman Z, Stein DJ, Seedat S. Predictors of acute stress disorder severity. Journal of affective disorders. 2013;149:277–81.

76. Whitman JB, North CS, Downs DL, Spitznagel EL. A prospective study of the onset of PTSD symptoms in the first month after trauma exposure. Annals of Clinical Psychiatry. 2013;25:163–72.

77. Mosaku K, Akinyoola A, Olasinde A, Orekha O. Predictors of posttraumatic stress in patients admitted to a trauma unit following road traffic accident (RTA). African Journal of Psychiatry. 2014;17:1–6.

78. Ogińska-Bulik N. Posttraumatic stress symptoms and posttraumatic growth in youth - Victims of road accidents. Psychiatria. 2014;11:49–58.

79. Heron-Delaney M, Kenardy J, Charlton E, Matsuoka Y. A systematic review of predictors of posttraumatic stress disorder (PTSD) for adult road traffic crash survivors. Inj. 2013;44:1413–22.

80. Sepahvand E, Khankeh H, Hosseini M, Akhbari B. Emotional Interaction in Road Traffic Injury: A Qualitative Study On People With Spinal Cord Injury. J Medicine Life. 2019;12:419–25.

81. Nutbeam T, Boylan M. ABC of Prehospital Emergency Medicine. London: Wiley Blackwell; 2013.

82. Govorova NV, Govorov VV, Govorov MV, Murasov MV. Temporary pelvic immobilization in providing assistance to victims with a high-energy combined trauma during transport. Novosti Khirurgii. 2016;24:151–6.

83. Floran AR, Alexandros NF, Eric RS, Olivier R, Pascal R, Nicolas JP, et al. Kendrick’s extrication device and unstable pelvic fractures: Should a trochanteric belt be added? A cadaveric study. Injury. 2AD;47:711–6.

84. Hospital PPAG. Prehospital management of spinal trauma: an evolution. Critical Care Nursing Quarterly. 1999;22:36–43.

85. Kim EG, Kuppermann N, Brown KM, Leonard J, Jaffe DM, Olsen CS. Method of spinal immobilization in children &lt;2 years old at risk for cervical spine injury (CSI). Pediatric Emergency Care. 2009;25:710–1.

86. Remsberg T, Widmeier K. Car seat safety...’Hold still: teaching pediatric immobilization techniques’ October, JEMS. JEMS: Journal of Emergency Medical Services. 2012;37:16–16.

87. Sporer. Why We Need to Rethink C-Spine Immobilization. EMS World. 2012;41:74–6.

88. Cowley A, Hague A, Durge N. Cervical spine immobilization during extrication of the awake patient: a narrative review. Eur J Emerg Med. 2017;24:158–61.

89. Cowley. Self-extrication in road traffic collisions: do we really need to cut the roof off? Journal of Paramedic Practice. 2014;6:584–90.

90. Clemency BM, Tanski CT, Chambers JG, O’Brien M, Knapp AS, Clark AJ, et al. Compulsory Use of the Backboard is Associated with Increased Frequency of Thoracolumbar Imaging. Prehospital emergency care : official journal of the National Association of EMS Physicians and the National Association of State EMS Directors. 2018;22:506–10.

91. Hauswald M. A re-conceptualisation of acute spinal care. Emergency Medicine Journal. 2013;30:720–3.

92. Sundstrøm T, Asbjørnsen H, Habiba S, Sunde GA, Wester K. Prehospital Use of Cervical Collars in Trauma Patients: A Critical Review. J Neurotrauma. 2014;31:531–40.

93. Benger J, Blackham J. Why do we put cervical collars on conscious trauma patients? Scand J Trauma Resusc Emerg Med. 2009;17:44.

94. Dunlap S, Clark D. Was I right?...standard of care during vehicle extrication. FireRescue Magazine. 1999;17:13–13.

95. Helm M, Lampl L, Hauke J, Bock KH. Accidental Hypothermia In Trauma Patients - is it Relevant to Prehospital Emergency Treatment. Anaesthesist. 1995;44:101–7.

96. Shek KC, Mak YK, Kam CW, Yau HH. Emergency medical team: An entrapped patient. Hong Kong Journal of Emergency Medicine. 2004;11:98–103.

97. Greenville S. Vehicle extrication: patient care as part of the process. Advanced Rescue Technology. 2004;7:37–40.

98. Cottingham, Thomson K. Use of ketamine in prolonged entrapment. Journal of Accident & Emergency Medicine. 1994;11:189–91.

99. Mackenzie R, Sutcliffe R. Pre-hospital care: the trapped patient. Journal of the Royal Army Medical Corps. 2000;146:39–46.

100. Puidupin A, Wiel E, Sfar SGE. Sedation and analgesia in emergency structure. Which sedation and/or analgesia for the entrapped patient? Annales Francaises D Anesthesie Et De Reanimation. 2012;31:347–52.

101. Cheng Y, Xue FS, Cui XL. Comparing the performance of different devices for endotracheal intubation in a simulated entrapped patient. European Journal of Emergency Medicine. 2014;21:150–1.

102. Daniel S, Thomas A, Christian H, Ulrich G. Comparison of airway management techniques for different access in a simulated motor vehicle entrapment scenario. European Journal of Emergency Medicine. 2AD;23:279–85.

103. Gaszynska E, Samsel P, Stankiewicz-Rudnicki M, Wieczorek A, Gaszynski T. Intubation by paramedics using the ILMA or AirTraq, KingVision, and Macintosh laryngoscopes in vehicle-entrapped patients: a manikin study. European Journal of Emergency Medicine. 2014;21:61–4.

104. Kaminska, Wieczorek W. Comparison of two types of laryngoscopy for face-to-face intubation of a patient entrapped in a vehicle. American Journal of Emergency Medicine. 2018;36:1898–9.

105. Wieczorek W, Gawel WB, Kaminska H. Ambu® AuraGain^TM^ laryngeal mask as a method of airway management of patient entrapped in vehicle. American journal of emergency medicine. 2019;37:171‐172.

106. Liu KP, Cheng Y, Xue FS, Li RP. Rational comparison of tracheal intubation with direct and indirect laryngoscopes in a simulated trapped manikin...Eur J Anaesthesiol. 2011 Dec;28(12):849-58. European Journal of Anaesthesiology (Cambridge University Press). 2012;29:496–9.

107. Martin AB, Lingg J, Lubin JS. Comparison of Airway Management Methods in Entrapped Patients: A Manikin Study. Prehospital Emergency Care. 2016;20:657–61.

108. Pap R, Loggerenberg C van. A comparison of airway management devices in simulated entrapment-trauma: a prospective manikin study. International Journal of Emergency Medicine. 2019;12.

109. Polk JD, Super DM, Kovach B, Russell S, Mancuso C, Fallon W. Comparison of the laryngeal mask airway versus blind endotracheal intubation in the simulated entrapped patient: a preliminary study. Air medical journal. 2001;20:21–2.

110. Raimann FJ, Tepperis DM, Meininger D, Zacharowski K, Schalk R, Byhahn C, et al. Comparing Four Video Laryngoscopes and One Optical Laryngoscope with a Standard Macintosh Blade in a Simulated Trapped Car Accident Victim. Emergency Medicine International. 2019;2019.

111. Schober P, Krage R, Groeningen DV, Loer S, Schwarte L. Inverse intubation of entrapped trauma casualties - A comparison of direct laryngoscopy, indirect optical laryngoscopy and video laryngoscopy in a simulated scenario. European Journal of Anaesthesiology. 2009;26:222.

112. Wolfgang AW, Andreas S, Robert S, Oliver S, Martin H, Jochen H. In a difficult access scenario, supraglottic airway devices improve success and time to ventilation. European Journal of Emergency Medicine. 12AD;22:374–6.

113. Wetsch WA, Carlitscheck M, Hinkelbein J. Video laryngoscopes in emergency medicine - A comparison using a simulated entrapped car accident victim. European Journal of Anaesthesiology. 2011;28:230–1.

114. Wolfgang AW, Martin C, Oliver S, Peter T, Martin H, Harald VG, et al. Success rates and endotracheal tube insertion times of experienced emergency physicians using five video laryngoscopes: a randomised trial in a simulated trapped car accident victim. European Journal of Anaesthesiology. 1AD;28:849–58.

115. Schyma B, Gobindram A, Joseph A, Kam JW, Shippey B, Chew K. Face to face airway management of the trapped driver. Anesthesia and Analgesia. 2016;123:708–9.

116. Castle N, Naguran S. Reflection: on the use of the ILMA in an entrapped patient. Emergency Medicine Journal. 2014;31:1014–5.

117. Hulme J, Perkins GD. Critically injured patients, inaccessible airways, and laryngeal mask airways. Emerg Med J. 2005;22:742.

118. Greene, Roden R, Hinchley G. The laryngeal mask airway. Two cases of prehospital trauma care. Anaesthesia. 1992;47:688–9.

119. Aplenc P. Decision making in airway management in prehospital emergency medicine regarding the use of alternative methods effecting the artificial airway. Resuscitation. 2010;81.

120. Pelinka LE, Thierbach AR, Reuter S, Mauritz W. Bystander trauma care--effect of the level of training. Resuscitation. 2004;61:289–96.

121. Heightman AJ. Impact of early care: first responder care often makes the difference. JEMS: Journal of Emergency Medical Services. 2011;36:14–14.

122. Bhalla K, Sriram V, Arora R, Ahuja R, Varghese M, Agrawal G, et al. The care and transport of trauma victims by layperson emergency medical systems: a qualitative study in Delhi, India. Bmj Global Health. 2019;4.

123. Reines HD, Bartlett RL, Chudy NE, Kiragu KR, McKnew MA. Is advanced life support appropriate for victims of motor vehicle accidents: The South Carolina Highway Trauma Projects. Journal of Trauma. 1988;28:563–70.

124. Gholipour C, Vahdati SS, Notash M, Miri SH, Ghafouri RR. Success rate of pre-hospital emergency medical service personnel in implementing pre hospital trauma life support guidelines on traffic accident victims. Turkiye Acil Tip Dergisi. 2014;14:71–4.

125. Cox J, Chapman TG. General practitioner attendance at emergencies notified to ambulance control. British medical journal (Clinical research ed). 1984;289:165–6.

126. Lampl L, Helm M, Weidringer JW, Bock KH. Prehospital management of entrapment trauma. Aktuelle Traumatologie. 1994;24:163–8.

127. Jaslow D, Barbera JA, Desai S, Jolly BT. An emergency department-based field response team: Case report and recommendations for a “go team.” Prehospital Emergency Care. 1998;2:81–5.

128. Høyer CCS, Christensen EF, Andersen NT. On-scene time in advanced trauma life support by anaesthesiologists. European Journal of Emergency Medicine. 2006;13:156–9.

129. Cohilas A, Hood J, Lowe B. Vehicle accident with entrapment: Call a physician to the scene! Fire Engineering. 2007;160:127–30.

130. Dissmann PD, Clerc SL. The experience of Teesside helicopter emergency services: doctors do not prolong prehospital on-scene times. Emerg Med J. 2007;24:59–62.

131. Hirano Y, Abe T, Tanaka H. Efficacy of the presence of an emergency physician in prehospital major trauma care: A nationwide cohort study in Japan. The American journal of emergency medicine. 2019;37:1605–10.

132. P. Byrne MPJ, Clay Mann PMSN, Mengtao Dai M, A. Mason MPS, Paul Karanicolas MP, Sandro Rizoli MP, et al. Association Between Emergency Medical Service Response Time and Motor Vehicle Crash Mortality in the United States. JAMA Surg. 2019;154:286–93.

133. Tobias Gauss M, François-Xavier Ageron MP, Marie-Laure Devaud M, Guillaume Debaty MP, Stéphane Travers M, Delphine Garrigue M, et al. Association of Prehospital Time to In-Hospital Trauma Mortality in a Physician-Staffed Emergency Medicine System. JAMA Surg. 2019;154:1117–24.

134. Nutbeam T, Fenwick R, Hobson C, Holland V, Palmer M. Extrication time prediction tool. Emergency Medicine Journal. 2015;32:401–3.

135. Burstein JL, Henry MC, Alicandro JM, McFadden K, Thode HC, Hollander JE. Evidence for and impact of selective reporting of trauma triage mechanism criteria. Academic Emergency Medicine. 1996;3:1011–5.

136. Santana JR, Martinez R. Accuracy of Emergency Physician Data-Collection in Automobile Collisions. Journal of Trauma-Injury Infection and Critical Care. 1995;38:583–6.

137. Lerner EB, Jeremy TC, Alan B, Richard DL, Manish NS, Robert AS, et al. EMS Provider Assessment of Vehicle Damage Compared with Assessment by a Professional Crash Reconstructionist. Prehospital emergency care. 8AD;15:483–9.

138. Stefanopoulos N, Vagianos C, Stavropoulos M, Panagiotopoulos E, Androulakis J. Deformations and intrusions of the passenger compartment as indicators of injury severity and triage in head-on collisions of non-airbag-carrying vehicles. Injury-International Journal of the Care of the Injured. 2003;34:487–92.

139. Reyero D, Alvarez N, Jean-Louis C, Bermejo B, Beaumont C, Belzunegui T. ABCDE...K for kinematics. The structural deformity index and severity in crash victims. Resuscitation. 2010;81.

140. Sasser SM, Hunt RC, Faul M, Sugerman D, Pearson WS, Dulski T, et al. Guidelines for field triage of injured patients: recommendations of the National Expert Panel on Field Triage, 2011. MMWR Recomm Rep; 2012.

141. Sasser SM, Hunt RC, Sullivent EE, Wald MM, Mitchko J, Jurkovich GJ, et al. Guidelines for field triage of injured patients. Recommendations of the National Expert Panel on Field Triage. MMWR Recomm Rep; 2009.

142. Kim SC, Jeon HJ, Lee KH, Choi HY, Noble J, Lee K. On-scene factors that predict severe injury of patients involved in frontal crashes of passenger cars. European Journal of Trauma and Emergency Surgery. 2016;1–8.

143. Henry MC, Hollander JE, Alicandro JM, Cassara G, Omalley S, Thode HC. Incremental benefit of individual American College of Surgeons trauma triage criteria. Academic Emergency Medicine. 1996;3:992–1000.

144. Davidson, Rivara FP, Mack CD, Kaufman R, Jurkovich GJ, Bulger EM. Validation of prehospital trauma triage criteria for motor vehicle collisions. Journal of Trauma & Acute Care Surgery. 2014;76:755–61.

145. Edmundson P, Roden-Foreman JW, Rainey EE, Ewing M, Rapier N, Funk GA, et al. Intrusion, ejection, and death in the compartment: Mechanism-based trauma activation criteria fail to identify seriously injured patients. Journal of the American College of Surgeons. 2017;225.

146. Kazuhide M, Konstantinos C, William K, Christy P, Deidre G, Demetrios D. Should we still use motor vehicle intrusion as a sole triage criterion for the use of trauma center resources? Injury. 10AD;47:235–8.

147. Simon BJ, Legere P, Emhoff T, Fiallo VM, Garb J. Vehicular Trauma Triage by Mechanism - Avoidance of the Unproductive Evaluation. Journal of Trauma-Injury Infection and Critical Care. 1994;37:645–9.

148. Loza A, Wilkie S, McClung C, Henderson SO. The value of mechanism of injury in predicting severity and outcome for victims of motor vehicle accidents. Academic Emergency Medicine. 2010;17.

149. Derek I, David CC, Federico EV. Motor Vehicle Intrusion Alone Does Not Predict Trauma Center Admission or Use of Trauma Center Resources. Prehosp Emerg Care. 1AD;15:203–7.

150. Ceklic E, Tohira H, Ball S, Brown E, Brink D, Bailey P, et al. Motor vehicle crash characteristics that are predictive of high acuity patients: an analysis of linked ambulance and crash data. Prehospital emergency care : official journal of the National Association of EMS Physicians and the National Association of State EMS Directors. 2020;1–13.

151. Cook MDCH, Muscarella MDP, Praba MDAC, Melvin MDWS, Martin MDLC. Reducing Overtriage Without Compromising Outcomes in Trauma Patients. Arch Surg. 2001;136:752–6.

152. Long WB, Bachulis BL, Hynes GD. Accuracy and relationship of mechanisms of injury, trauma score, and injury severity score in identifying major trauma. American Journal of Surgery. 1986;151:581–4.

153. Stuke LE, Duchesne JC, Greiffenstein P, Mooney JL, Marr AB, Meade PC, et al. Not all mechanisms are created equal: a single-center experience with the national guidelines for field triage of injured patients. The journal of trauma and acute care surgery. 2013;75:140–5.

154. Lerner, Shah MN, Cushman JT, Swor RA, Guse CE, Brasel K, et al. Does mechanism of injury predict trauma center need? Prehospital Emergency Care. 2011;15:518–25.

155. Palanca S, Taylor DM, Bailey M, Cameron PA. Mechanisms of motor vehicle accidents that predict major injury. Emergen Med. 2003;15:423–8.

156. Lowe DK, Oh GR, Neely KW, Peterson CG. Evaluation of injury mechanism as a criterion in trauma triage. American Journal of Surgery. 1986;152:6–10.

157. Leichtle, Poulos NG, Whelan J, Aboutanos M. Field Triage of Motor Vehicle Crashes: Which Factors Predict High Injury Severity? American Surgeon. 2019;85:764–7.

158. Fullerton Z, Donald GW, Cryer HG, Lewis CE, Cheaito A, Cohen M, et al. Trauma System Overtriage: Are We on Track? American Surgeon. 2014;80:960–5.

159. Lerner EB, Badawy M, Cushman JT, Drendel AL, Fumo N, Jones CMC, et al. Does Mechanism of Injury Predict Trauma Center Need for Children? Prehosp Emerg Care. 2020;25:1–8.

160. Susan LE, Michael LN, Kristy BA, Michael RE, Flaura KW. Passenger compartment intrusion as a predictor of significant injury for children in motor vehicle crashes. Journal of Trauma. 2AD;66:504–7.

161. Newgard CD, Lewis RJ, Jolly BT. Use of out-of-hospital variables to predict severity of injury in pediatric patients involved in motor vehicle crashes. Annals of Emergency Medicine. 2002;39:481–91.

162. Ryb GE, Dischinger PC. Improving trauma triage using basic crash scene data. 2011. p. 337–46.

163. Schulman CI, Wilbur V, Leibowitz B, Labiste L, Perdeck E, Bahouth G, et al. The Scenescore for Improved Pre-Hospital Triage of Motor-Vehicle Crash Victims. Annual Proceedings / Association for the Advancement of Automotive Medicine. 1AD;51:49–60.

164. Augenstein J, Perdeck E, Stratton J, Digges K, Steps J, Bahouth G. Validation of the urgency algorithm for near-side crashes. Annual proceedings / Association for the Advancement of Automotive Medicine Association for the Advancement of Automotive Medicine. 2002;46:305–14.

165. Ayoung-Chee P, Mack CD, Kaufman R, Bulger E. Predicting severe injury using vehicle telemetry data. The journal of trauma and acute care surgery. 2013;74:190.

166. Pal C, Hirayama S, Sangolla N, Manoharan J, Kulothungan V. A new approach in improving traffic accident injury prediction accuracy. Int J Automot Eng. 2017;8:179–85.

167. Fenwick R, Nutbeam T. Medical vs. true physical traffic collision entrapment. J Paramedic Pract. 2018;10:158–62.

168. Lars W, Boye HT, Kjell K, Andreas SP. Rapid extrication from a car wreck. Injury. 1AD;35:739–45.

169. Fattah S, Johnsen AS, Andersen JE, Vigerust T, Olsen T, Rehn M. Rapid extrication of entrapped victims in motor vehicle wreckage using a Norwegian chain method - cross-sectional and feasibility study. BMC emergency medicine. 2014;14:14.

170. Bucher J, Santos FD, Frazier D, Merlin M. Rapid Extrication versus the Kendrick Extrication Device (KED): Comparison of Techniques Used After Motor Vehicle Collisions. WestJEM. 2015;16:453–8.

171. Shafer JS, Naunheim RS. Cervical spine motion during extrication: a pilot study. The western journal of emergency medicine. 2009;10:74–8.

172. Engsberg JR, Standeven JW, Shurtleff TL, Eggars JL, Shafer JS, Naunheim RS. Cervical Spine Motion during Extrication. JEM. 2013;44:122–7.

173. Gabrieli A, Nardello F, Geronazzo M, Marchetti P, Liberto A, Arcozzi D, et al. Cervical Spine Motion During Vehicle Extrication of Healthy Volunteers. Prehosp Emerg Care. 2019;24:1–14.

174. David H, Lars S, Jeronimo ONW, Berthold G, Adrian R, Paul AG, et al. An explorative, biomechanical analysis of spine motion during out-of-hospital extrication procedures. Inj. 2AD;51:185–92.

175. Dixon M, O’Halloran J, Cummins NM. Biomechanical analysis of spinal immobilisation during prehospital extrication: a proof of concept study. Emergency Medicine Journal. 2014;31:745–9.

176. Dixon M, O’Halloran J, Hannigan A, Keenan S, Cummins NM. Confirmation of suboptimal protocols in spinal immobilisation? Emerg Medicine J Emj. 2015;32:939–45.

177. Sackett DL, Rosenberg WMC, Gray JAM, Haynes RB, Richardson WS. Evidence based medicine: what it is and what it isn’t. Bmj. 1996;312:71.

**Acknowledgements:** An adapted version of this systematic scoping review was submitted as a chapter in The Development of Evidence-Based Guidelines to Inform the Extrication of Casualties Trapped in Motor Vehicles following a Collision, towards Doctor of Philosophy (Phd) In Emergency Medicine, University of Cape Town 2022. Supervisors: Dr Willem Stassen, Professor Jason Smith, Professor Lee Wallis

**Supplementary Text 2: The Extrication In Trauma (EXIT) Project**

The Extrication In Trauma (EXIT) Project aimed to develop evidence-based guidance for extricating patients trapped in motor vehicles following collisions, challenging the long-held paradigm of absolute spinal movement minimisation during extrication.

The project employed an evidence-based medicine (EBM) framework across multiple studies:

Retrospective Cohort Studies: Analysis of the UK Trauma Audit and Research Network (TARN) database demonstrated that trapped patients had significantly higher mortality (8.9% vs 5.0%, p<0.001) and injury severity scores compared to non-trapped patients . Importantly, the rate of spinal cord injuries that might influence extrication technique was remarkably low (0.7%) [[1]](https://www.zotero.org/google-docs/?lHuWNI).

Sex-disaggregated analysis revealed females were more frequently trapped (15.8% vs 9.4%, p<0.0001) and demonstrated different injury patterns, with higher rates of spinal and pelvic injuries compared to males [[2]](https://www.zotero.org/google-docs/?qwEl6u). Age-related analysis showed older patients had excess mortality associated with entrapment but similar potential for self-extrication as younger patients [[3]](https://www.zotero.org/google-docs/?egoakU).

Biomechanical Analysis: Four studies utilising inertial motion units assessed spinal movement during various extrication techniques [[4–7]](https://www.zotero.org/google-docs/?Dk2HI2). Self-extrication produced the smallest anterior-posterior cervical spine movement (2.6mm), whilst rapid extrication resulted in the largest (6.21mm). The differences between self-extrication and other methods were significant (p<0.001), though minimal differences existed between roof removal, b-post rip and rapid removal techniques [[5]](https://www.zotero.org/google-docs/?WozsDk).

Patient Experience: Qualitative interviews identified communication as the primary theme influencing patient experience. Positive experiences were associated with clear communication, companionship, and planned follow-up, while negative experiences correlated with communication failures and loss of autonomy [[8]](https://www.zotero.org/google-docs/?MEDbEY).

Consensus Development: A Delphi study involving 60 experts nominated by key stakeholders achieved consensus on 91 statements covering multiple domains of extrication practice [[9]](https://www.zotero.org/google-docs/?MVZq6g). These formed the basis for new evidence-based principles adopted by national stakeholders.

**References:**

[1. Nutbeam T, Fenwick R, Smith J, Bouamra O, Wallis L, Stassen W. A comparison of the demographics, injury patterns and outcome data for patients injured in motor vehicle collisions who are trapped compared to those patients who are not trapped. Scand J Trauma Resusc Emerg Med. 2021;29:17.](https://www.zotero.org/google-docs/?ALlWTU)

[2. Nutbeam T, Weekes L, Heidari S, Fenwick R, Bouamra O, Smith J, et al. Sex-disaggregated analysis of the injury patterns, outcome data and trapped status of major trauma patients injured in motor vehicle collisions: a prespecified analysis of the UK trauma registry (TARN). BMJ Open. 2022;12:e061076.](https://www.zotero.org/google-docs/?ALlWTU)

[3. Nutbeam T, Kehoe A, Fenwick R, Smith J, Bouamra O, Wallis L, et al. Do entrapment, injuries, outcomes and potential for self-extrication vary with age? A pre-specified analysis of the UK trauma registry (TARN). Scand J Trauma Resusc Emerg Med. 2022;30:14.](https://www.zotero.org/google-docs/?ALlWTU)

[4. Nutbeam T, Fenwick R, May B, Stassen W, Smith JE, Wallis L, et al. The role of cervical collars and verbal instructions in minimising spinal movement during self-extrication following a motor vehicle collision - a biomechanical study using healthy volunteers. Scand J Trauma Resusc Emerg Med. 2021;29:108.](https://www.zotero.org/google-docs/?ALlWTU)

[5. Nutbeam T, Fenwick R, May B, Stassen W, Smith JE, Bowdler J, et al. Assessing spinal movement during four extrication methods: a biomechanical study using healthy volunteers. Scand J Trauma Resusc Emerg Med. 2022;30:7.](https://www.zotero.org/google-docs/?ALlWTU)

[6. Nutbeam T, Fenwick R, May B, Stassen W, Smith J, Shippen J. Maximum movement and cumulative movement (travel) to inform our understanding of secondary spinal cord injury and its application to collar use in self-extrication. Scand J Trauma Resusc Emerg Med. 2022;30:4.](https://www.zotero.org/google-docs/?ALlWTU)

[7. Nutbeam T, Fenwick R, May B, Stassen W, Smith JE, Bowdler J, et al. A biomechanical study to compare spinal movement in a healthy volunteer during extrication between “chain cabling” and “roof off” methods of extrication. Injury. 2022;53:3605–12.](https://www.zotero.org/google-docs/?ALlWTU)

[8. Nutbeam T, Brandling J, Wallis LA, Stassen W. Understanding people’s experiences of extrication while being trapped in motor vehicles: a qualitative interview study. BMJ Open. 2022;12:e063798.](https://www.zotero.org/google-docs/?ALlWTU)

[9. Nutbeam T, Fenwick R, Smith JE, Dayson M, Carlin B, Wilson M, et al. A Delphi study of rescue and clinical subject matter experts on the extrication of patients following a motor vehicle collision. Scand J Trauma Resusc Emerg Med. 2022;30:41.](https://www.zotero.org/google-docs/?ALlWTU)

**Supplementary Text 3: Attendees Briefing Document**

In preparation for: 15^th^ April 2024

### Introduction:

The purpose of this meeting is to:

- Reach consensus on the clinical and operational translation to practice of consensus guidance on extrication reached through the EXIT project
- Reach consensus on additional areas of extrication related clinical and operational practice where:
  - The translation to practice of the EXIT project has identified gaps in current guidance
  - The original consensus document lacked clarity, would benefit from refinement or should be updated
- Resolve any additional questions raised by attendees

Planned format of the day:

09:00 - 10:00 Arrivals, coffee and networking

10:00 - 10:30 Introduction to the EXIT project

10:30 - 11:00 JRCALC and EXIT

11:00 - 11:30 NOG and EXIT

11:30 - 12:00 Spinal Injury, major trauma and EXIT

12:00 - 12:30 A legal perspective

12:30 - 13:30 Lunch

13:30 - 15:30 Translation of EXIT to Practise: Consensus, Challenges and Solutions

15:30 - 16:00 Wrap up and next steps

### Background:

The EXIT project was established to consider post-collision response, research and translation.

In June 2022, the EXIT project published consensus findings on the extrication of patients following a motor vehicle collision. This consensus process was supported by Subject Matter Experts from the National Fire Chiefs Council (NFCC), the United Kingdom Rescue Organisation (UKRO), the National HEMS Research & Audit Forum (NHRAF), the College of Paramedics (CoP), the Pre-Hospital Trainee Operated Research Network (PHOTON) and the Faculty of Prehospital Care (FPHC).

Principles:

- Operational and clinical team members should work together to develop a bespoke patient centred extrication plan with the primary focus of minimising entrapment time.

- Independent of actual or suspected injuries, patients should be handled gently. A focus on absolute movement minimisation is not justified.

- When clinicians are not available, FRSs should where necessary assess patients, deliver clinical care and make and enact extrication plans (including self-extrication)^1^

- Self-extrication or minimally assisted extrication should be the standard ‘first line’ extrication for all patients who do not have contraindications. The contraindications are:

§ An inability to understand or follow instructions,

§ Injuries or baseline function that prevents standing on at least one leg, (specific injuries include: unstable pelvic fracture, impalement, bilateral leg fracture)

- All patients with evidence of injury should be considered time-dependent and their entrapment time should be minimised.

- Incidents where a patient may require disentanglement are complex and associated with a high morbidity and mortality. A senior FRS and clinical response should attend such instances^2^.

Clinical care during entrapment:

o Can be delivered by FRS or clinical services^1^.

o Should be limited to necessary critical interventions to expedite safe extrication^3^

o Rescuers should be aware that clinical observations may prolong entrapment time and as such should be kept to the minimum.

o FRS and clinical personnel should be aware of the physical and observable signs of patient deterioration and if identified should make this known to the responsible clinician.

Immobilisation:

o Longboards are an extrication device and should not be used beyond the extrication phase.

o Kendrick Extrication Devices prolong extrication time, and their use should be minimised.

o Pelvic slings should not be applied to patients until they have been extricated.

o Cervical collars should only be used following assessment and should be loosened or removed following extrication.

Patient focused extrication:

o Build a connection with patients, explain actions, and use their name.

o Where appropriate, reassure patients as to the safety of their co-occupants and others involved in the incident (including animals)

o Provide an ‘extrication buddy’.

o Allow communication with family members or other close contacts

o Rescue teams should not publish extrication related imagery to social media or other outlets.

o Minimise the ability of the public to view the accident, take photographs or record videos. Provide education to this effect.

On initial call to Emergency Services:

o Attempt to clarify entrapment status

o Attempt to identify patients who require disentanglement (and dispatch an appropriate priority senior^2^ response)

o A standard multi-agency MVC trauma message should be developed to ensure the correct resources are deployed.

- Multi-professional datasets should be developed with patient and public engagement and should include entrapment status, entrapment time, injuries, extrication approach, clinical care

**Agreed nomenclature for categories of patient:**

- Not injured
- Minor injuries (evidence of energy transfer but no evidence of time-dependent injury)
- Major injury (currently stable but should be assumed to be time-dependent)
- Time critical injured (Time critical due to injury; use fastest route of extrication)
- Time critical hazard (e.g. secondary to fire or other hazard)

**Footnotes:**

1 FRS clinical care should be standardised and delivered with appropriate training and clinical governance oversight.

2 A senior or enhanced clinical and operational response should be dispatched. This may include enhanced / critical care and will benefit from further consideration.

3 In-car interventions may include the administration of tranexamic acid, analgesia and oxygen. Interventions may include the management of compressible haemorrhage and decompression of suspected tension pneumothorax. Patients who require volume (fluid or blood product) resuscitation are likely to have time critical injuries and their removal from the vehicle should be prioritised. In the small number of patients who cannot be released quickly then ‘in vehicle’ fluids and /or blood products may be required.

### Integration into clinical and operational practice:

The EXIT project team has worked closely with Joint Royal Colleges Ambulance Liaison Committee (JRCALC) and the National Operational Guidance (NOG) team to support the development of guidance which facilitates the delivery of the EXIT principles.

Anecdotally there have been reports of successful integration of the EXIT principles into practice and many “good news” stories where this has led to positive patient outcomes. However, (as expected) integration and adoption has not been seamless and there is more work to do.

The purpose of this meeting is to:

- Reach consensus on the clinical and operational translation to practice of consensus guidance reached through the EXIT project
- Reach consensus on additional areas of extrication related clinical and operational practice where:
  - The translation to practice of the EXIT project has identified gaps in current guidance
  - The original consensus document lacked clarity, would benefit from refinement or should be updated

Subjects identified for discussion and consensus:

1. Case study review
2. Empowerment of FRS personnel to risk stratify and deliver self-extrication and define limits of this practice.
3. Empowerment of lay persons on scene to deliver self-extrication and define limits of this practice
4. Communication on scene / development of shared language / tools
5. Location of patients post-extrication
6. Training requirements needs
7. Additional steps for translation to practise

We ask that if you are participating in the consensus finding exercise (all clinical and operational personnel) that you are familiar with these and come prepared to vote and/ or discuss. You will be asked your opinions prior to the meeting, areas not reaching consensus will be discussed at the meeting.

### Case study review

These case studies have emerged through the preparation of the FPHC consensus process and through feedback to the EXIT team.

A: Is self-extrication appropriate if the casualty is experiencing neck or back pain.

Background: Minor / moderate soft tissue injury to the neck and back is common following a MVC.

Suggested response / actions:

1. Apply the “U STEP OUT” tool or similar
2. Give analgesia (pain relief) if required / available
3. Encourage self-extrication or assisted self-extrication


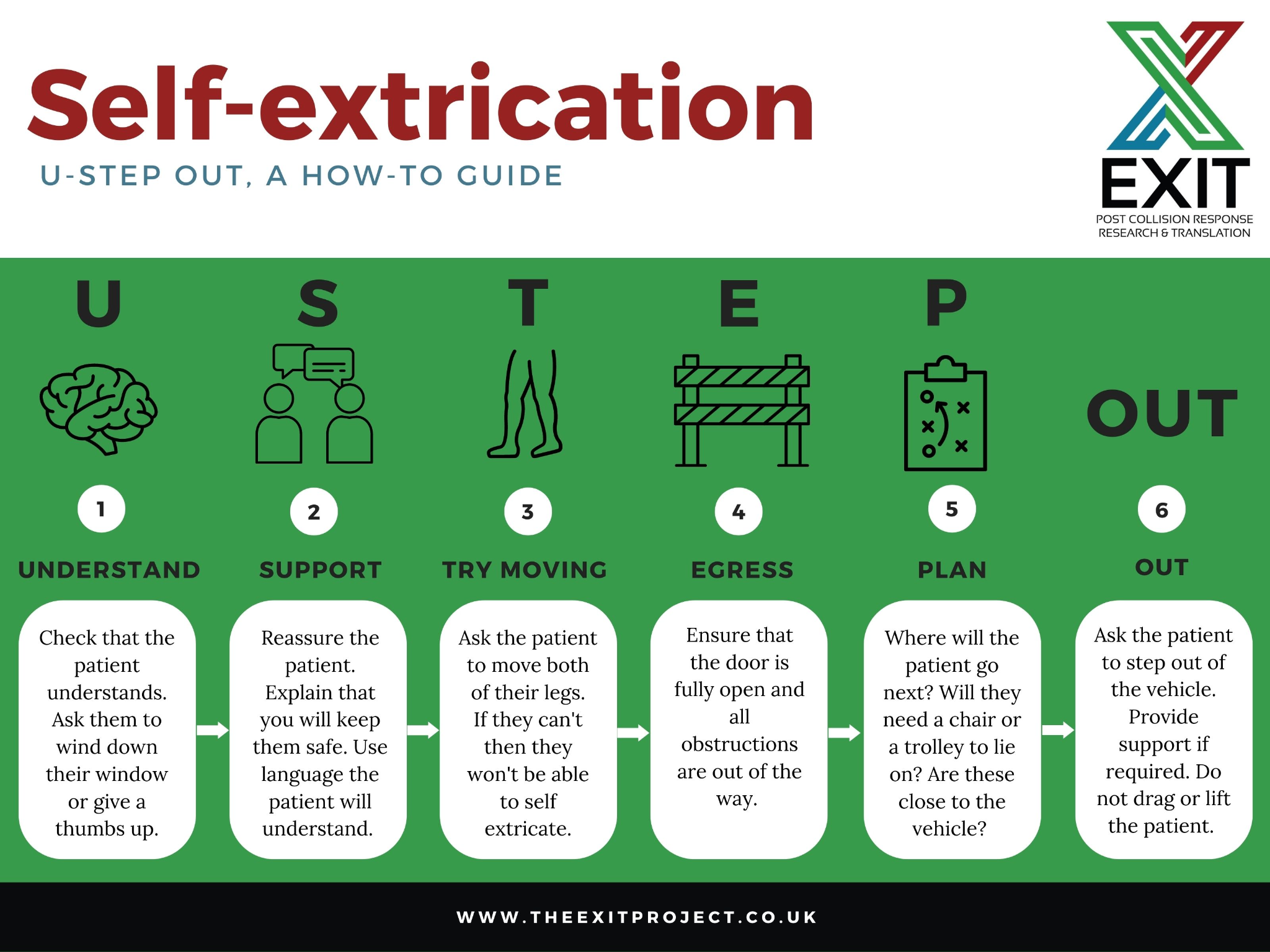


B: Is self-extrication appropriate if there are soft neurological signs (e.g. non-dermatomal tingling)

Background: Some patients following a MVC will experience symptoms which may be considered by the rescuer as neurological in nature. These may include pins and needles, a sensation of tingling or other altered sensation.

Suggested response / actions:

1. Confirm there is no “motor” deficit through a simple screening test - for example the patient is able to move their hands and feet.
2. Facilitate self-extrication if there are no contraindications

Suggested decision maker: Responder trained in assessment and self-extrication. NOT for lay decision making.

C: Is self extrication appropriate if central cord signs?

Background: A small number of extrication MVC’s are associated with spinal cord injury. This may present as a ‘central cord’ syndrome. In this situation the upper limbs may have hard neurological signs (eg unable to move) but the lower limbs may be preserved. As such these patients may be able to participate in self-extrication.

Additional:

- Half of patients with spinal cord injury will have other major injuries which may compromise the patient's ability to minimise secondary harm to their spinal cord
- Optimising clinical care for a patient with spinal cord injury will be compromised by prolonged entrapment times and this may lead to worse long-term sequelae
- Identifying injury is challenging and major injuries are often missed on clinical assessment, even by expert clinicians
- Central cord syndrome patients may deteriorate on weight bearing

Suggested response / actions:

1. Confirm there is no “motor” deficit of the lower limbs
2. Primary: Facilitate self-extrication with hand-on support throughout
3. Secondary: Aim for rapid extrication with gentle patient handling (not absolute movement minimisation)

Suggested decision maker: Responder trained in assessment and self-extrication. NOT for lay decision making. Requires 2 + trained responders to deliver assisted self-extrication in this group.

D: Actions if neurological signs evolve during self-extrication

Background: A small number of extrication MVC’s are associated with spinal cord injury. Weight bearing may reveal neurological signs which were not present prior to weight bearing.

Additional:

- Half of patients with spinal cord injury will have other major injuries which may compromise the patient's ability to minimise secondary harm to their spinal cord
- Optimising clinical care for a patient with spinal cord injury will be compromised by prolonged entrapment times and this may lead to worse long-term sequelae
- Identifying injury is challenging and major injuries are often missed on clinical assessment, even by expert clinicians

Suggested response / actions:

1. Provide immediate support / assisted self-extrication
2. Continue with self-extrication if possible
3. If not possible: Aim for rapid extrication with gentle patient handling (not absolute movement minimisation)

E: Actions if hard neurological signs present on initial assessment (e.g. patient unable to move legs)

Background: A small number of extrication MVC’s are associated with spinal cord injury. Patient’s with hard neurological signs are unlikely to be able to participate in self-extrication.

Additional:

- Half of patients with spinal cord injury will have other major injuries which may compromise the patient's ability to minimise secondary harm to their spinal cord
- Optimising clinical care for a patient with spinal cord injury will be compromised by prolonged entrapment times and this may lead to worse long-term sequelae
- Identifying injury is challenging and major injuries are often missed on clinical assessment, even by expert clinicians

Suggested response / actions:

1. Aim for rapid extrication with gentle patient handling (not absolute movement minimisation)

We invite participants to submit additional case studies for consideration prior to the meeting on the 15th April

### Empowerment of FRS personnel to risk stratify and deliver self-extrication and define limits of this practice.

###

### **Background:**

### Outcomes from the EXIT project include:

1. All patients with evidence of injury should be considered time-dependent and their entrapment time should be minimised.
2. When clinicians are not available, FRSs should where necessary assess patients, deliver clinical care and make and enact extrication plans (including self-extrication)^1^
3. Self-extrication or minimally assisted extrication should be the standard ‘first line’ extrication for all patients who do not have contraindications, which are:

§ An inability to understand or follow instructions,

§ Injuries or baseline function that prevents standing on at least one leg, (specific injuries include: unstable pelvic fracture, impalement, bilateral leg fracture)

Suggested response / actions:

### FRS personnel should be enabled (with appropriate training and governance structures) to deliver self-extrication and assisted self-extrication across all patients. FRS should ensure that this assessment and delivery skill-set is widely available to their patients.

1. The U-STEP-OUT algorithm can be used by all FRS personnel (this is focused at a smaller sub-group of patients)

###

###

### Empowerment of lay persons on scene to deliver self-extrication and define limits of this practice

### **Background:**

### Outcomes from the EXIT project include:

1. All patients with evidence of injury should be considered time-dependent and their entrapment time should be minimised.

Suggested response / actions:

1. The U-STEP-OUT algorithm in various forms (app / visual prompt / telephone guided) can be used by lay members of the public and other responding professional groups (e.g. police) following further translational work.

###

### Communication on scene / development of shared language / tools

**Background:**

Failures in communication and / or a shared mental model of extrication priorities are a common area of feedback to the EXIT project.

EXIT outcome, agreed nomenclature for categories of patient:

- Not injured
- Minor injuries (evidence of energy transfer but no evidence of time-dependent injury)
- Major injury (currently stable but should be assumed to be time-dependent)
- Time critical injured (Time critical due to injury; use fastest route of extrication)
- Time critical hazard (e.g. secondary to fire or other hazard)

Suggested response / actions:

A standardised, national, multi-professional communication tool should be developed, disseminated and appropriate training and oversight provided to ensure adoption into practice.

We invite participants to submit their ideas in relation to this tool prior to the meeting on the 15th April.

### Location of patients post-extrication

**Background:**

A regular question asked in relation to implementation of EXIT is where patients should be physically located and where responsibility lies following self-extrication.

Suggested response / actions:

1. All patients should be moved to an environmentally safe location (e.g. away from an active highway / under appropriate cover)
2. Patients who self-declare as uninjured or minor injuries and able to meet their own needs should be identified as not requiring further clinical assessment and their details passed to NHS Ambulance service control centre.
3. Communication between FRS and clinical response prior to arrival should occur and look to:
4. Optimise patient outcome / experience
5. Optimise the use and availability of clinical and operational resource

We invite participants to submit their ideas in relation to this scenario prior to the meeting on the 15th April.

### Training requirements needs

###

**Background:**

### This subject area should be considered in context of those considered above. Clarity is required on what training materials are required to optimise adoption of the EXIT project outcomes.

Suggested response / actions:

1. The U-STEP-OUT algorithm in various forms (app / visual prompt / telephone guided) can be used by lay members of the public and other responding professional groups (e.g. police) following further translational work.
2. The U-STEP-OUT algorithm can be used by operational and clinical response without additional training.
3. A multi-disciplinary training package should be developed and made available which empowers clinicians and FRS to deliver self-extrication and assisted self-extrication.

### Additional steps for translation to practise

These will be discussed at the meeting on the 15th.
